# Research progress on S-palmitoylation modification mediated by the ZDHHC family in glioblastoma

**DOI:** 10.3389/fcell.2024.1413708

**Published:** 2024-11-05

**Authors:** Beiyan Tang, Wei Kang, Qiang Dong, Zhenwei Qin, Lei Duan, Xianjun Zhao, Guoqiang Yuan, Yawen Pan

**Affiliations:** ^1^ The Second Medical College of Lanzhou University, Lanzhou, Gansu, China; ^2^ Department of Neurosurgery, Second Hospital of Lanzhou University, Lanzhou, Gansu, China; ^3^ Key Laboratory of Neurology of Gansu Province, Lanzhou University, Lanzhou, Gansu, China; ^4^ Academician Workstation, The Second Hospital of Lanzhou University, Lanzhou, Gansu, China

**Keywords:** glioblastoma, zDHHC family, S-palmitoylation, molecular mechanism, treatment strategy

## Abstract

S-Palmitoylation has been widely noticed and studied in a variety of diseases. Increasing evidence suggests that S-palmitoylation modification also plays a key role in Glioblastoma (GBM). The zDHHC family, as an important member of S-palmitoyltransferases, has received extensive attention for its function and mechanism in GBM which is one of the most common primary malignant tumors of the brain and has an adverse prognosis. This review focuses on the zDHHC family, essential S-palmitoyltransferases, and their involvement in GBM. By summarizing recent studies on zDHHC molecules in GBM, we highlight their significance in regulating critical processes such as cell proliferation, invasion, and apoptosis. Specifically, members of zDHHC3, zDHHC4, zDHHC5 and others affect key processes such as signal transduction and phenotypic transformation in GBM cells through different pathways, which in turn influence tumorigenesis and progression. This review systematically outlines the mechanism of zDHHC family-mediated S-palmitoylation modification in GBM, emphasizes its importance in the development of this disease, and provides potential targets and strategies for the treatment of GBM. It also offers theoretical foundations and insights for future research and clinical applications.

## 1 Introduction

S-palmitoylation is a highly conserved post-translational lipid modification widely present in eukaryotes. It plays crucial roles in various physiological processes by influencing protein structure, localization, transport, and function (Tabaczar et al., 2017). Based on the different acyl acceptors, protein palmitoylation can be classified into S-palmitoylation, N-palmitoylation, and O-palmitoylation (Zhang Y. et al., 2021). Among these modifications, S-palmitoylation involves the attachment of palmitic acid (PA) to target proteins via thioester bonds (Sobocińska et al., 2018). Due to the specificity of thioester bonds, this modification is a valuable reversible modification (Qu et al., 2021). S-palmitoylated proteins are predominantly membrane proteins, especially transmembrane and peripheral membrane proteins (Charollais and Van Der Goot, 2009). Upon modification, target proteins undergo changes in hydrophobicity and membrane-binding ability, thereby altering protein structure, localization, transport, and function. The combination of S-palmitoylation modification and its reverse reaction—depalmitoylation—leads to periodic changes in the structure and function of modified proteins, aligning with specific biological functions of individuals (Li et al., 2022).

**FIGURE 1:**
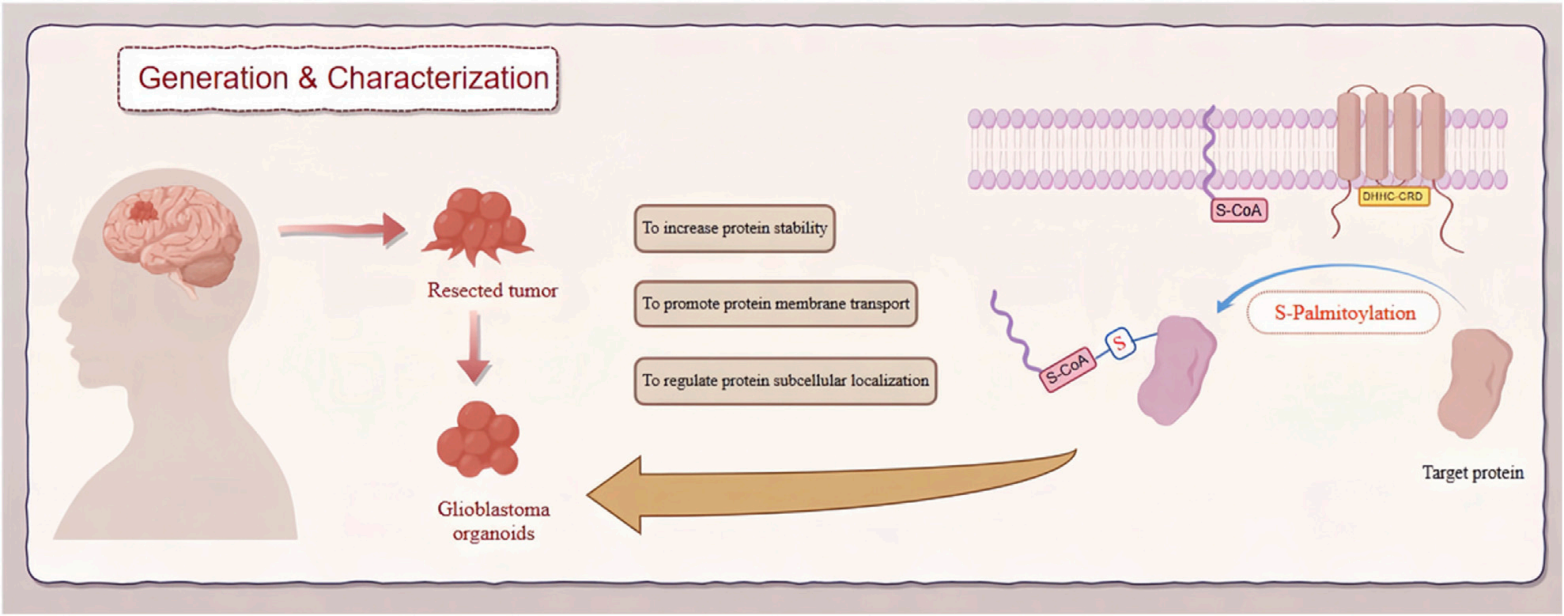
Role of zDHHC-mediated S-palmitoylation in GBM. This diagram depicts the involvement of zDHHC enzymes in S-palmitoylation, a post-translational modification, and its effects on the pathogenesis of GBM. zDHHC enzymes facilitate the addition of palmitoyl groups to specific substrate proteins, altering their localization, stability, and function. These modifications influence key signaling pathways that drive GBM progression and malignancy.

As the most malignant primary tumor of the brain, Glioblastoma has long been a challenge for neurologists, oncologists, clinicians and so on Shergalis et al., 2018. Understanding its pathogenesis has been a focal and challenging aspect of scientific research. The significant role of zDHHC-mediated palmitoylation in GBM is increasingly recognized, particularly in key proteins associated with its occurrence and progression (Chen et al., 2020a). Increasing evidence suggests that palmitoylation or palmitoyltransferases could become novel targets for cancer therapy (Chen et al., 2018). This review comprehensively analyzes how zDHHC-mediated S-palmitoylation modification regulates the occurrence and development of GBM (Figure 1). Additionally, it explores the potential of developing protein S-palmitoylation-targeted drugs for more precise personalized therapy.

## 2 S-palmitoylation

S-palmitoylation is a reversible protein lipid modification that widely exists in eukaryotic cells and participates in regulating downstream gene transcription, expression, and signal transduction, thereby influencing cellular activities (Resh, 2013). Discovered in the 1980s, palmitoylation is catalyzed by the protein acyltransferase (PAT) family, which attaches palmitic acid (PA), a 16-carbon saturated fatty acid, to cysteine residues on target proteins (Schmidt et al., 1979; Chen et al., 1985; Mitchell et al., 2006). PA, which is one of the important substrates involved in palmitoylation modification, is the most common saturated fatty acid in the human body and constitutes 20%–30% of total fatty acids and can be obtained from dietary sources like meat and dairy (Carta et al., 2017; Carta et al., 2015) or synthesized endogenously through *de novo* lipogenesis (DNL) from other metabolites (Song et al., 2018; Kersten, 2001). Of note, PA plays essential physiological roles, serving as a component of cell membranes in the form of phospholipids and sphingolipids, providing energy through β-oxidation, or being esterified into triglycerides for lipid storage (Carta et al., 2017). Changes in PA concentration directly affect the efficiency and function of palmitoylation (Pei et al., 2016; Wang et al., 2023). Upon modification, the hydrophobicity and affinity of target proteins for the cell membrane surface increase.

Unlike irreversible lipid modifications such as myristoylation or prenylation, which involve amide or thioether bonds, respectively, S-palmitoylation forms thioester bonds (Robert and Vagner, 2018; Yuan et al., 2020a). These bonds are less stable due to the larger atomic radius and weaker bonding energy of sulfur compared to oxygen (Ashenhurst, 2015; van Bergen et al., 2016). Additionally, sulfur atoms are prone to oxidation and reduction reactions, leading to the instability of thioester bonds and enabling proteins to cycle between palmitoylated and depalmitoylated forms, thus spatio-temporal control of protein function can be conferred (Ko and Dixon, 2018; Wang et al., 2020). Depalmitoylation, the reverse reaction of palmitoylation, is catalyzed by palmitoyl-protein thioesterases (PPTs), including acyl-protein thioesterases (APT1/2), palmitoyl-protein thioesterases (PPT1/2), or proteins containing α/β hydrolase domains (ABHD17A/B/C) (Hornemann, 2015). Overall, depalmitoylation serves as an essential complement to palmitoylation, necessary for protein function and cellular processes. The dynamic balance between palmitoylation and depalmitoylation allows cells to regulate protein activity, stability, and localization to adapt to different cellular environments and physiological needs. Many key proteins in cellular signaling pathways undergo palmitoylation, and depalmitoylation can alter their activity and stability, thereby affecting signal transduction. Through depalmitoylation, cells can timely adjust the activity of signaling pathways to adapt to different cellular stimuli.

## 3 ZDHHC

The enzymes catalyzing S-palmitoylation modification, known as PATs, are referred to as DHHC-PATs (Mitchell et al., 2006). In most cases, these enzymes contain a cysteine-rich domain (CRD) comprising 51 amino acids. Within the CRD, there’s a highly conserved catalytic domain called the aspartate-histidine-histidine-cysteine (DHHC) motif, which is crucial for maintaining the palmitoylation activity of the PAT molecule (Tabaczar et al., 2017; Gottlieb et al., 2015). The DHHC domain serves as the catalytic center of the enzyme, and mutations in this domain can weaken the palmitoyl transfer capability (Ko and Dixon, 2018; Resh, 2006a). Additionally, the CRD contains two zinc-binding sites that coordinate with two Zn^2+^ ions which do not directly participate in protein catalysis play a vital role in maintaining the integrity and functionality of the domain (Zmuda and Chamberlain, 2020; Stix et al., 2020). Due to the presence of the zinc finger DHHC domain, this class of PATs is also referred to as zinc finger DHHC domain-containing palmitoyl transferases (zDHHC).

Up to present, There are 23 zDHHC genes identified in the human genome, named zDHHC1-24 (excluding zDHHC10) (Ko and Dixon, 2018). These zDHHC proteins constitute the zDHHC protein family, primarily responsible for the catalytic activity of palmitoyl transferases (Linder and Deschenes, 2007). These enzymes are mainly localized in the endoplasmic reticulum (ER), Golgi apparatus, with a smaller fraction found on the plasma membrane (PM), mitochondria and perinuclear regions (Chen et al., 2021; Greaves and Chamberlain, 2011; Jansen and Beaumelle, 2022). The localization of DHHC-PATs partly depends on structure and motif. For example, lysine-based sorting signals on the sequences of DHHC4 and DHHC6 enable them to form KXX and KKXX motifs, thus guiding them to the ER membrane (Gorleku et al., 2011).

Structurally, the molecular structure of zDHHC proteins which as a type of polytopic integral membrane protein consists of an N-terminal domain facing the cytoplasm, 4-7 transmembrane domains (TMDs), and a C-terminal domain also facing the cytoplasm (Ohno et al., 2006). N-terminal and C-terminal sequences exhibit significant diversity, mediating protein-protein interactions and facilitating acyl transfer processes (Jiang et al., 2018; Malgapo and Linder, 2021). The TMDs collectively form a cavity resembling a tent, facilitating the binding of fatty acyl chains (Rana et al., 2018). The DHHC domain, crucial for maintaining catalytic activity, is located between the second and third TMDs (Jiang et al., 2018; Rana et al., 2019). Besides the DHHC motif, other conserved motifs such as Asp-Pro-Gly (DPG), Thr-Thr-x-Glu (TTxE), and PaCCT have been reported (Mitchell et al., 2006). The second serine in the TTxE motif can directly interact with the aspartate of the DHHC motif to form hydrogen bonds, while Asn266 in the PaCCT motif utilizes its hydrogen bonding capability to assist inside chain amide formation (Rana et al., 2018). This suggests that these conserved residues may also participate in substrate protein recognition and catalysis processes by making key contacts with the DHHC domain (Rana et al., 2018).

Some zDHHC proteins require interaction with auxiliary factors to form complexes, such as the DHHC9/GCP16 complex (Swarthout et al., 2005) and the DHHC6/Selk complex (Fredericks et al., 2014). Additionally, certain zDHHC enzymes require palmitoylation by other family members to initiate S-palmitoylation cascades, thereby regulating substrate palmitoylation (Abrami et al., 2017; Plain et al., 2020). Specific zDHHC molecules may exhibit unique substrate binding preferences, as evidenced by zDHHC13 and zDHHC17’s strong selectivity for synaptic-related proteins (Rodenburg et al., 2017; Zhang and Hang, 2017). Notably, zDHHC13 and zDHHC17 contain an ankyrin repeat domain (AR) within their N-terminal domains (Lemonidis et al., 2017), facilitating membrane localization and enhancing the palmitoylation activity of other zDHHC enzymes (Lemonidis et al., 2014; Lemonidis et al., 2015). These findings underscore the complex and efficient structure-function relationship within the zDHHC family, providing insights into the mechanism underlying protein palmitoylation.

## 4 Mechanisms of S-palmitoylation

Although 23 zDHHCs have been identified to catalyze protein S-palmitoylation, the specific process remains unclear (Yuan et al., 2024). Previous studies have found that in most cases, zDHHCs catalyze substrate S-palmitoylation through a two-step process, known as the ping-pong kinetic mechanism (Rana et al., 2019; Roth et al., 2002). First, zDHHC proteins undergo auto-palmitoylation, where the cysteine residue in the DHHC-CRD domain of zDHHC protein covalently binds with palmitoyl coenzyme A to form an acyl-enzyme intermediate (Malgapo and Linder, 2021). Subsequently, the crucial second step reaction occurs, where zDHHC proteins catalyze the transfer of the palmitoyl group from their own binding site to the cysteine thiol group of the protein substrate. Simultaneously, zDHHC proteins revert to their initial state, and the protein substrate forms an unstable thioester bond, completing the palmitoylation modification (Rana et al., 2018; Jennings and Linder, 2012; Chamberlain and Shipston, 2015; Salaun et al., 2020). The aforementioned reaction mechanisms apply to the catalytic processes of most zDHHC molecules (Stix et al., 2020). However, there is still insufficient evidence to determine whether zDHHC13, 17, and 19 can catalyze auto-palmitoylation or not. Further investigation may be needed to explore common mechanisms underlying zDHHC catalyzed palmitoylation modification and whether auto-palmitoylation does not occur in all zDHHCs (Lemonidis et al., 2014; Verardi et al., 2017).

Most proteins undergo palmitoylation on multiple amino acid residues, with reports suggesting up to 5-6 individual palmitoylated cysteine residues in some proteins (Zhang et al., 2018). While the above two-step kinetic mechanism applies to the majority of proteins, it has been observed that some proteins undergo auto-palmitoylation independently of the zDHHC protein family. Examples include yeast proteins (Swf1 and Pfa4) (Das et al., 2021), myelin protein P0 (Bharadwaj and Bizzozero, 1995) and others, which possess acyl-CoA within their structures. Due to the unique characteristics of their structures, some of these proteins exhibit intrinsic “enzyme-like” activities and can directly bind with palmitoyl-CoA to undergo auto-palmitoylation (Smotrys and Linder, 2004; Chan et al., 2016).

In conclusion, our comprehension of the mechanisms by which ZDHHC and APT enlist their protein substrates remains incomplete (Rana et al., 2018; Verardi et al., 2017; Lee et al., 2022). Data from the SwissPalm database reveals that palmitoylation encompasses over 2,400 mammalian proteins, as evidenced by findings from more than 100 proteomics and biochemical studies (Blanc et al., 2015). These proteins encompass integral membrane proteins, peripheral membrane proteins, cytoplasmic signaling proteins, and transcription factors. Oncoproteins such as NRAS, KRAS4A, HRAS, epidermal growth factor receptor (EGFR), and p53 depend on S-palmitoylation cycling to modulate their localization, activity, or interaction partners (Tate et al., 2019).

## 5 Functions of S-palmitoylation

From a biochemical perspective, the binding of the lipid molecule palmitic acid can enhance the hydrophobicity of specific domains of target proteins (Jiang et al., 2018). Additionally, due to the instability of thioester bonds, palmitoylation and depalmitoylation can rapidly switch, acting as a protein switch or modulator similar to protein ubiquitination or phosphorylation (Blaskovic et al., 2013). Changes in hydrophobicity/hydrophilicity may affect various properties and functions of proteins. It can modulate the affinity of protein molecules for membrane structures, thereby determining protein localization (Levental and Lyman, 2023). Through different localizations, protein-protein interactions (PPI) may be altered, leading to activation or inhibition of downstream signaling pathways, affecting processes such as gene replication, transcription, expression, or protein secretion (Zhang Z. et al., 2021; Pei et al., 2022; Lee et al., 2021).

Palmitoylation modification plays a crucial role in the pathological and physiological functions of key proteins in signaling pathways such as RAS, MET, STING, EGFR and Hedgehog, etc. (Runkle et al., 2016; Lin et al., 2017). Taking the Ras protein family as an example, newly synthesized Ras proteins undergo palmitoylation modification during transport to the Golgi apparatus to enter the secretory pathway and transfer to the PM After activation at the PM, depalmitoylation occurs, weakening the protein’s binding to the membrane, leading to its transport back to the Golgi apparatus (Shahinian and Silvius, 1995; Busquets-Hernández and Triola, 2021). The dynamic palmitoylation of Ras regulates its cycling between the PM and Golgi apparatus, preventing nonspecific residence on the PM and facilitating the transmission of activated Ras signals downstream (Rocks et al., 2005). Moreover, there is evidence suggesting that EGFR signaling can be both promoted and inhibited by its palmitoylation. This might be due to the presence of multiple palmitoylation sites, which are regulated by different ZDHHC proteins and have distinct functional effects (Bollu et al., 2015). Palmitoylation also regulates the activity of G protein-coupled receptors, including the melanocortin-1 receptor (MC1R), which drives melanin production and DNA repair following UV exposure (Chen S. et al., 2017). Therefore, impaired palmitoylation of MC1R increases the risk of melanoma. Additionally, palmitoylation is involved in regulating the tumor microenvironment, including crucial processes such as angiogenesis and immune evasion (Lee et al., 2021; Pan and Chen, 2022). S-palmitoylation imparts different characteristics to proteins by attaching palmitic acid molecules, thereby affecting protein hydrophobicity, structural stability, localization, migration between membrane regions, and interactions with effectors, enzyme activity, protein storage, and more.

S-palmitoylation is also associated with resistance to cancer treatments. For example, ZDHHC2-mediated palmitoylation of mitochondrial acylglycerol kinase (AGK) increases sunitinib resistance in renal cell carcinoma by activating the AKT–mTOR signaling pathway (Sun et al., 2023). Similarly, ZDHHC16-mediated palmitoylation of PCSK9 induces sorafenib resistance in cancer through activation of the PI3K–AKT pathway (Sun et al., 2022).

## 6 Glioblastoma

Glioblastoma (GBM) is a common malignant tumor originating from the central nervous system, which remains essentially incurable. Arising from neural glial cells, GBM can occur in various parts of the brain, accounting for approximately 51% of all malignant tumors of the brain. According to statistics, the incidence of GBM is 5 per 100,000 individuals, with a rising trend annually. While the affected population primarily comprises middle-aged and elderly individuals, GBM can occur across all age groups, including children (Xiao et al., 2023). Despite the standard treatment regimen for GBM, which involves maximal safe surgical resection followed by adjuvant therapies such as temozolomide (TMZ) chemotherapy, adjunctive radiotherapy, and tumor-treating fields (TTF) therapy, the median survival period post-diagnosis remains merely 8 months, with a 5-year survival rate of less than 7% (Zhong et al., 2021; Ostrom et al., 2022). Additionally, the majority of GBM patients experience tumor recurrence (Jiang et al., 2020; Daniel et al., 2022). The high recurrence rate may be attributed to the presence of dormant tumor-seeding cells located distant from the initial tumor or tumor cells infiltrating the normal brain parenchyma at the tumor’s periphery (Darmanis et al., 2017; Johnson et al., 2014).

GBM, also known as glioblastoma multiforme, was classified by the World Health Organization (WHO) in 2016 into IDH-wildtype and IDH-mutant subtypes based on the mutation status of isocitrate dehydrogenase (IDH) (Louis et al., 2016). Due to the closer resemblance of IDH-mutant GBM to anaplastic astrocytoma, some scholars have proposed renaming IDH-mutant GBM as astrocytoma, IDH-mutant, grade 4 (White et al., 2020). The 2021 WHO classification defines GBM as lacking IDH mutation, along with alterations related to telomerase reverse transcriptase (TERT) promoter mutation, epidermal growth factor receptor (EGFR) amplification, chromosome 10 copy number loss, and chromosome 7 copy number gain (Louis et al., 2021).

As a typical solid tumor, GBM exhibits characteristics of sustained cell proliferation, resistance to cell death, continuous angiogenesis, increased cellular invasion and metastasis. These features are accompanied by genomic instability and mutations, altered cellular metabolism, replicative immortality, sustained inflammation, evasion of growth suppression, and immune suppression (Hanahan, 2022; Verdugo et al., 2022). The mechanisms underlying its occurrence involve multiple aspects such as genetics, epigenetics, and transcriptomics, ultimately leading to significant alterations in crucial signaling pathways. The entire process can be summarized as follows: (Tabaczar et al., 2017): External signaling molecules activate signaling pathways via membrane proteins; (Zhang Y. et al., 2021); Protein kinases activate signals from the membrane to the nucleus; (Sobocińska et al., 2018); Transcription factors activate effector genes, resulting in cellular biological effects (Blanc et al., 2015; Lothrop et al., 2013; Nakada et al., 2011).

Current research has revealed several key factors contributing to the occurrence and development of GBM, including gene mutations (Brennan et al., 2013), tumor microenvironment (TME) (Quail and Joyce, 2017; Erices et al., 2023) and aberrant signaling pathways (Anido et al., 2010; Stommel et al., 2007). These processes are typically controlled by various oncogenes and/or tumor suppressor genes, many of which undergo post-translational modifications (PTMs) such as phosphorylation (Huang et al., 2021; Wang et al., 2021), methylation (Li et al., 2023; Leske et al., 2023), acetylation (Li et al., 2024; Liu X. et al., 2023), ubiquitination (Chang et al., 2023) and so on, which to some extent affect protein localization, stability, and function (Fhu and Ali, 2021). Protein lipidation is a significant and diverse class of post-translational modifications (PTMs) that involves the covalent attachment of specific lipid molecules to various amino acid residues on target proteins (Jiang et al., 2018; Liu et al., 2021). Lipidated proteins typically exhibit a stronger affinity for non-polar structures, such as lipid bilayers, influencing the localization, diffusion, and interactions of these modified proteins (Chen et al., 2018; Lanyon-Hogg et al., 2017; Burnaevskiy et al., 2015). It is widely recognized that this modification can regulate various biological processes in eukaryotic cells, including cell division, differentiation, and immune responses (Resh, 2006b). Most protein lipidation modifications are considered irreversible, including N-myristoylation (Lin et al., 2012), S‐farnesylation (Kmiec et al., 2021), O‐palmitoylation (Zou et al., 2011), and N‐palmitoylation (Chen et al., 2004), etc. In contrast, protein S-palmitoylation is a reversible post-translational modification (PTM) (Jiang et al., 2018; Linder and Jennings, 2013). This characteristic allows the modified proteins to cycle between palmitoylated and depalmitoylated forms, making it essential for understanding how protein palmitoylation affects the function of individual proteins in both normal and cancer cells (Martin et al., 2011; Won and Martin, 2018).

## 7 zDHHC and GBM

**FIGURE 2:**
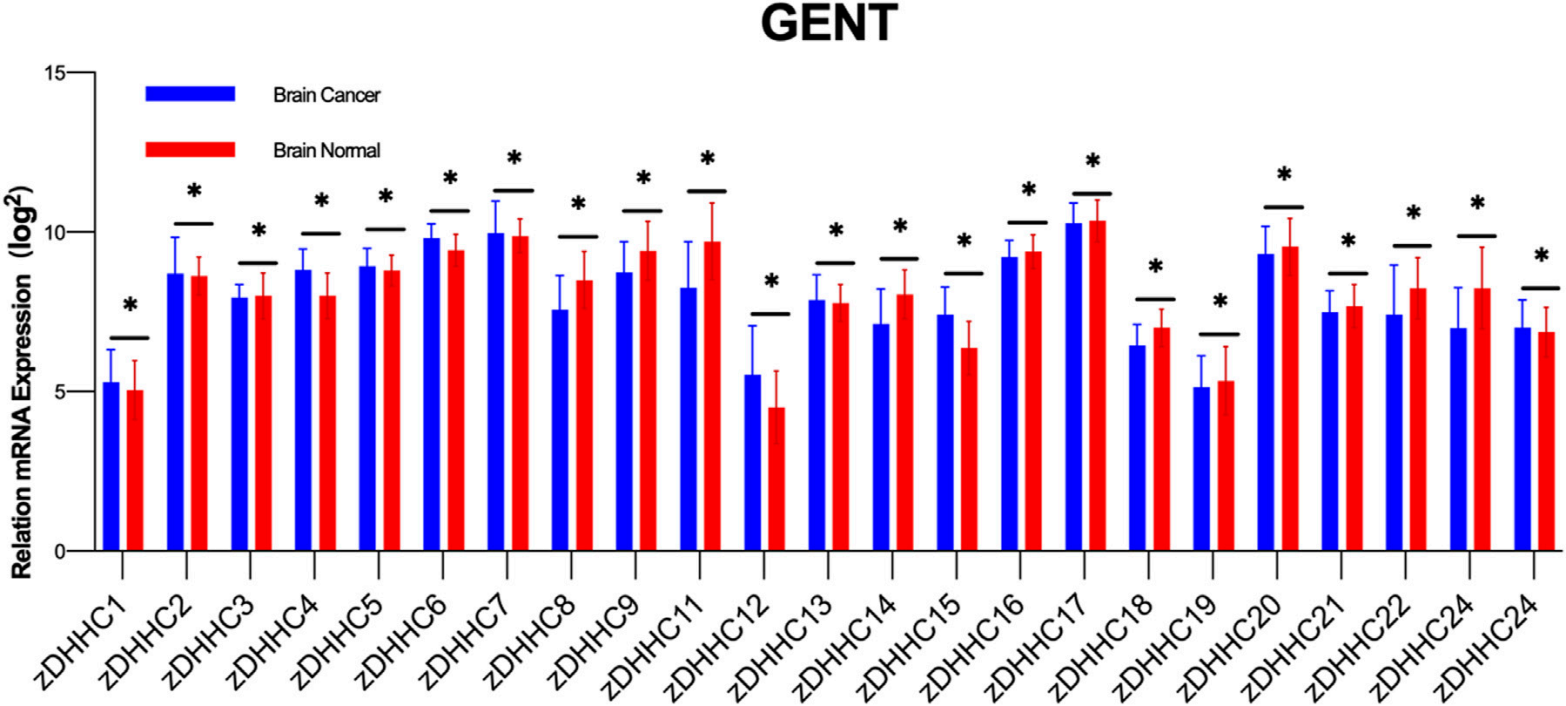
Differential Expression of zDHHC Genes in Normal Brain Tissue and Glioblastoma. The figure shows the differential expression of various zDHHC genes between normal brain tissues (n = 873) and glioblastoma tumor tissues (n = 3439) as analyzed from the GENT database. Multiple unpaired t-tests were conducted to compare the expression levels of each zDHHC gene in the two types of tissues. The bar graph shows the mean values with error bars representing the standard deviation (SD). The results indicate significant statistical differences in the expression of zDHHC genes between normal and tumor tissues, highlighting their potential role in the pathogenesis of glioblastoma. Each group showed significant *p*-values, denoting strong evidence against the null hypothesis of no difference in expression levels.

Similar to lipid imbalance situations, palmitoylation not only participates in numerous physiological processes but also associates with various diseases, including neurological disorders, viral infections, cardiovascular diseases, immune system disorders, and the onset and progression of tumors (Mesquita et al., 2021; Ramos et al., 2023; Chong et al., 2023; Zhou et al., 2022). The importance of protein palmitoylation in tumorigenesis has been a subject of attention and research over the past decade. Palmitoylation influences multiple aspects of cancer, including cancer cell proliferation and survival, invasion and metastasis, and anti-tumor immunity (Ko and Dixon, 2018; Jin et al., 2021; Tang et al., 2022a). The crucial role of zDHHC-mediated palmitoylation in GBM is also increasingly recognized, particularly in key proteins associated with its occurrence and progression (Chen et al., 2020a). In our preliminary study, we compared the expression of various zDHHC molecules in normal brain tissue and brain tumors by querying the GENT database (Figure 2). Accumulating evidence suggests that palmitoylation or palmitoyltransferases may serve as novel targets for cancer therapy (Liu et al., 2020).

Understanding the cellular signaling and molecular mechanisms of palmitoylation modification, as well as its pathological effects, is crucial. Here, we will review the latest research to unveil the mechanisms by which zDHHC-mediated S-palmitoylation modification regulates protein function in the occurrence and progression of GBM. Finally, we aim to explore opportunities and new strategies for therapeutic intervention targeting protein palmitoylation (Table 1).

**TABLE 1:** Target proteins, pathways and biological roles ZDHHC protein-mediated protein palmiylation

ZDHHCs	Target proteins	Pathways	Actions	Biological effects
zDHHC3	PD-L1, Cys272	Immune evasion pathway	Increases the stability and intracellular expression of PD-L1 through palmitoylation, inhibiting T cell activity	Regulates immune evasion and survival of tumor cells
zDHHC4	GSK-3B,-	GSK-3B/EZH2/STAT3 pathway	Modulates the phosphorylation status of GSK-3ẞ through palmitoylation, affecting self-renewal and drug resistance of GSCs	Participation in GSCs self-renewal and regulation of resistance to TMZ
zDHHC5	EZH2, Cys571 and Cys576	EZH2/S21 signaling pathway	Modulates the phosphorylation status of EZH2 and membrane localization of FAK1 through palmitoylation, affecting cell growth and migration	Regulates self-renewal of glioblastoma stem cells and epithelial- mesenchymal transition
zDHHC7	STAT3,-	JAK2/STAT3 signaling pathway	Affects membrane localization and activation of STAT3 through palmitoylation, regulating cell signaling	Involved in sustained activation of STAT3 signaling pathway in glioblastoma
zDHHC9	GLUT1, Cys207	Glucose metabolism pathway	Promotes membrane localization of GLUT1 through palmitoylation, increasing glucose uptake and cell proliferation	Regulates energy metabolism and proliferation of GBM cells
zDHHC15	gp130, -	IL-6/STAT3 signaling pathway	Modulates gp130 signaling by palmitoylation, inhibiting IL-6/STAT3 signaling pathway	Involved in regulation of glioblastoma stem cell sphere formation, proliferation, and growth
zDHHC16	SETD2	DNA damage repair pathway	Modulates DNA damage repair signaling by affecting SETD2 palmitoylation	Involved in regulation of DNA damage repair signaling in GBM
zDHHC17	MAP2K4, -	JNK/p38 signaling pathway	Activates JNK/p38 pathway through interaction with MAP2K4, regulating development and progression of GBM	Involved in regulation of malignant characteristics and growth of GBM
zDHHC18	BMI1 and RNF144A,-	BMII signaling pathway	Affects state transition of GSCs by modulating BMI1 ubiquitination levels	Regulates state transition of GBM stem cells
zDHHC23	BMI1 and RNF144A,-	BMII signaling pathway	Affects stability of BMI1 in susceptible neural stem cells by increasing BMI1 ubiquitination levels	Regulates state transition of GBM stem cell
zDHHC19	Smad3,-	TGF-B signaling pathway	Promotes Smad3 activation through palmitoylation, regulating TGF-ẞ signaling pathway	Involved in regulation of GBM cell tendency and mesenchymal subtype

### 7.1 zDHHC3

GBM is considered a “cold tumor,” where tumor cells, microglia, macrophages, T cells, and myeloid-derived suppressor cells (MDSCs) collectively form an immunosuppressive microenvironment (Da Ros et al., 2018; Lim et al., 2018). Programmed Death Ligand-1 (PD-L1) is expressed on the surface of GBM cells, which, upon binding to Programmed Death-1 (PD-1) on T cell surfaces, resists T cell cytotoxicity, ultimately leading to tumor immune evasion (Yao et al., 2021; Yang Y. et al., 2019; Freeman et al., 2000; Feng et al., 2023). Tang et al. demonstrated the colocalization and physical interaction between ZDHH3 and PD-L1 in GBM cell lines using immunofluorescence and co-immunoprecipitation in their glioma research (Tang W. et al., 2022). zDHHC3-mediated palmitoylation modification plays a crucial role in regulating tumor immune evasion and tumor cell survival. Through palmitoylation modification of PD-L1 protein (Cys272), zDHHC3 inhibits PD-L1 monoubiquitination, preventing its delivery to lysosomes for degradation, thereby increasing PD-L1 expression levels within cells. This results in increased surface expression of PD-L1 on tumor cells, which, by binding to PD-1, inhibits T cell activity, reducing tumor cell immune clearance (Yao et al., 2019). Blocking PD-L1 palmitoylation modification pharmacologically promotes PD-L1 lysosomal degradation, thereby activating anti-tumor immune responses and enhancing T cell cytotoxicity. Additionally, palmitoylation modification by zDHHC3 affects the intracellular distribution of PD-L1, depleting its intracellular pool, making anti-PD-L1 therapy more effective (Tang W. et al., 2022). Therefore, zDHHC3-mediated palmitoylation modification regulates the stability and function of PD-L1, influencing tumor immune evasion and survival. Current antibody therapies mainly target surface expression of PD-L1, while targeting PD-L1 palmitoylation simultaneously reduces its expression levels on the cell membrane and within cells. This strategy sensitizes tumor cells to T cell cytotoxicity more effectively, thereby more efficiently inhibiting tumor growth (Kinney et al., 2017).

### 7.2 zDHHC4

Glycogen Synthase Kinase-3β (GSK-3β) belongs to the serine/threonine protein kinase family and is primarily localized in the cytoplasm, with distribution in the nucleus and mitochondria as well. Apart from its initial discovery in regulating the activity of glycogen synthase (GS), GSK-3β also influences the structure and function of various signaling proteins and transcription factors, contributing to tumor formation and progression. Depending on the different molecular modifications it undergoes, GSK-3β exhibits diverse effects on tumor cells (T et al., 2016; He et al., 2020). Zhao et al. (C et al., 2022) found that zDHHC4 catalyzes the palmitoylation modification of GSK-3β, followed by phosphorylation of EZH2 at S21. This process further regulates the phosphorylation and methylation of STAT3. Through the GSK-3β-EZH2-STAT3 axis, it is involved in the self-renewal of Glioblastoma Stem Cells (GSCs) and the development of resistance to TMZ (a chemotherapy drug) in GBM. Additionally, this modification affects the interaction between GSK3β and AKT and p70S6K, thereby regulating their phosphorylation status. This discovery reveals the critical role of GSK-3β palmitoylation modification in the regulation of tumor stem cells, providing a new theoretical basis for understanding and intervening in tumor development.

### 7.3 zDHHC5

The ZDHHC5 gene is located in an unstable chromosomal region and is upregulated in breast cancer and lung adenocarcinoma (Tian et al., 2015). Chen et al. (Chen X. et al., 2017) found that the p53 gene controls the transcriptional activation of the ZDHHC5 promoter in GBM, suggesting an association between ZDHHC5 expression, p53 regulation, and tumorigenesis. Furthermore, it was discovered for the first time that in GBM, both the mRNA and protein levels of ZDHHC5 increase with tumor grading, correlating with increased p53 mutation frequency. Further functional studies revealed that mutant p53 can physically interact with the transcription factor NF-Y, forming an enhanced functional complex independent of the type of p53 mutation, leading to aberrant upregulation of ZDHHC5. Additionally, overexpression of ZDHHC5, along with mutations in KRAS, TERT, and p53 oncogenes, is sufficient to trigger comprehensive and rapid malignant transformation of GBM. EZH2 is a histone methyltransferase targeting H3K27 and is thus considered a suppressor of tumor suppressor genes. When ZDHHC5 catalyzes the palmitoylation modification of EZH2 at Cys571 and Cys576, it affects the phosphorylation level of EZH2 at the S21 position, and the levels of these two modifications are inversely correlated. Palmitoylation is a necessary condition for EZH2 localization to the Golgi apparatus, while phosphorylation occurs at the Golgi apparatus. Decreasing the levels of ZDHHC5 and EZH2 palmitoyltransferase significantly inhibits the growth of glioma tumors (Tang et al., 2022c). ZDHHC5 can inhibit the expression of other pluripotency-related transcription factors by inhibiting EZH2 activation, thus preventing the self-renewal of GBM stem cells. In a recent study, Wang et al. (Wang et al., 2024) discovered that ZDHHC5 can also catalyze the S-palmitoylation of the tumor-related protein Focal Adhesion Kinase 1 (FAK1), thereby enhancing FAK’s localization on the cell membrane and promoting epithelial-mesenchymal transition (EMT) of cells. EMT is a crucial process in tumor progression, enabling tumor cells to acquire invasive and migratory capabilities (Pastushenko and Blanpain, 2019; Thiery et al., 2009). Therefore, ZDHHC5-mediated palmitoylation modification of FAK may promote the development and metastasis of GBM by promoting the EMT process. This discovery provides important clues for understanding the molecular mechanisms of tumor development and offers new targets for therapeutic strategies targeting this modification process. Overall, the upregulation of ZDHHC5 in GBM is associated with p53 mutations and plays a role in influencing the self-renewal of GSCs and tumorigenesis. These findings suggest that ZDHHC5 may be a potential therapeutic target for treating p53-mutant GBM.

### 7.4 zDHHC7

Signal transducer and activator of transcription 3 (STAT3) is a transcription factor located in the cytoplasm, whose activation by JAK, MAPK, or mTOR kinases can lead to phosphorylation of tyrosine or serine residues in the C-terminal domain of the STAT3 protein and its dimerization, thereby activating STAT3 dimers to enter the nucleus and initiate transcription of target genes (Becker and Wilting, 2019). Typically, STAT3 activation is transient and modulated. However, in tumor cells, 50%–90% of tumor cells exhibit sustained activation (LI et al., 2019) and in 66%–83% of GBM, the STAT3 pathway is constitutively activated (Carro et al., 2010; Kim et al., 2013). Moreover, the level of STAT3 phosphorylation is closely related to tumor grading, with significant differences observed between low-grade and high-grade tumors (Mizoguchi et al., 2006).

Advancements in research on palmitoylation mediated by zDHHC7 in GBM have demonstrated its significant role in the transcription factor STAT3. Studies have shown that cysteine 108 of STAT3 is palmitoylated by zDHHC7, which promotes the membrane localization of STAT3 and phosphorylation of JAK2, thereby affecting STAT3 activation and cellular signaling (Zhang et al., 2020). Additionally, it has been found that APT2 selectively depalmitoylates p-STAT3, promoting its nuclear translocation. This research reveals the crucial role of the palmitoylation-depalmitoylation cycle in regulating STAT3 activation, providing important clues for understanding the role of palmitoylation in the pathogenesis of GBM. These findings may provide a basis for the development of new therapeutic strategies. For example, therapeutic strategies targeting zDHHC7 and APT2 may help intervene in the STAT3 signaling pathway, thereby impacting the development and treatment of GBM.

### 7.5 zDHHC9

Glucose transporter 1 (GLUT1) belongs to the glucose transporter protein family and is responsible for the transmembrane uptake of glucose under normal conditions (Hg et al., 2002; Mueckler et al., 1985). It is widely expressed in various tissues, with the highest expression levels observed in endothelial cells of barrier tissues such as red blood cells and the blood-brain barrier (Uldry and Thorens, 2004). In the central nervous system, GLUT1 plays a crucial role in endothelial cells and astrocytes, facilitating glucose uptake in astrocytes and glucose transport across the blood-brain barrier, making it considered the primary energy transporter in the brain.

A prominent characteristic of cancer is altered metabolism (Pavlova et al., 2022) and GLUT1 is aberrantly expressed in various cancers, including lung cancer, brain cancer, breast cancer, and bladder cancer (Ganapathy et al., 2009; Macheda et al., 2005). In the case of GBM, studies have shown high levels of GLUT1 expression in GBM cells to meet their high energy demands for glucose uptake (Mg et al., 2009; Komaki et al., 2019). DHHC9-mediated S-palmitoylation of GLUT1 is crucial for maintaining its localization on the cytoplasmic membrane. This palmitoylation promotes glucose uptake, glycolysis rate, and lactate production in GBM cells, thereby facilitating cell proliferation and tumor formation (Zhang Z. et al., 2021). This discovery is correlated with patient prognosis, highlighting the importance of zDHHC9 in regulating GLUT1 function in GBM.

### 7.6 zDHHC15

Glycoprotein 130 (gp130) is a glycoprotein located on the cell membrane and is one of the signaling receptor subunits of the interleukin-6 (IL-6) family (Cron et al., 2016; Wagener et al., 2014). It directly regulates signaling pathways such as STAT, MAPK, and PI3K/AKT and activates the SOCS negative feedback regulatory mechanism (Nogueira-Silva et al., 2013). As a crucial protein mediating cell survival, abnormal stabilization and overexpression of gp130 is closely associated with tumor progression. In normal cells, the level of gp130 protein is intricately regulated through various mechanisms, including ubiquitin-dependent degradation, endocytosis, and Caspase-induced protein cleavage. Specifically, the tetraspanin CD9 forms a complex with gp130, reducing its ubiquitination and maintaining high levels of gp130 in GSCs, ensuring sustained activation of STAT3 (Shi et al., 2017).

zDHHC15 plays a role by palmitoylating the IL-6 receptor subunit gp130, inhibiting the IL-6/STAT3 signaling pathway. Through a positive feedback mechanism, zDHHC15 effectively suppresses GSCs’ sphere formation, cell proliferation, and growth (Fan et al., 2021a). Local anesthetics such as lidocaine, bupivacaine, mepivacaine, or ropivacaine can disrupt the transcription of ZDHHC15, reducing the palmitoylation level of gp130 and its localization on the cell membrane, thereby inhibiting the activation of the IL-6/STAT3 signal. This study emphasizes the crucial role of zDHHC15 in regulating the IL-6/STAT3 signaling pathway, which has a beneficial impact on the biological behavior of GSCs. Furthermore, the expression of ZDHHC15 in GBM is positively correlated with tumor grade, and high expression levels are associated with GSC self-renewal (Liu Z-Y. et al., 2023). Therefore, ZDHHC15 is not only an important regulatory factor for GSCs but also a potentially useful biomarker for GBM diagnosis and prognosis.

### 7.7 zDHHC16

Fan et al. (Fan et al., 2022) demonstrated that the ZDHHC16/SETD2/H3K36me3 signaling axis is inactivated in EGFR-altered GBM. Specifically, ZDHHC16 is significantly downregulated in GBM compared to normal brain tissue, which is closely associated with changes in EGFR. These events lead to the activation of p53, causing cells to arrest at the G1/S checkpoint. Additionally, in EGFR-amplified GBM, ionizing radiation-induced DNA damage affects DNA damage repair signaling, involving reduced palmitoylation of SETD2 and methylation of its target H3K36.

### 7.8 zDHHC17

ZDHHC17 is primarily localized in the Golgi apparatus, and its deficiency leads to arrest at the G2/M transition, along with an increase in the proportion of cells with dense Golgi. Studies have shown that ZDHHC17 is upregulated in GBM and interacts with MAP2K4 through its N-terminal signal transduction and protein-protein interaction ankyrin domain, forming a signaling module that activates the JNK/p38 pathway, thereby regulating the malignant development and progression of GBM (Chen et al., 2020b). This finding suggests the importance of the ZDHHC17-MAP2K4 signaling pathway in GBM and provides clues for further understanding the pathogenesis of GBM. Importantly, the JNK/p38 activation promoted by ZDHHC17 is independent of PAT, and glioma cells with suppressed ZDHHC17 expression are insensitive to 2-BP inhibition. This discovery highlights the importance of ZDHHC17 in regulating the JNK/p38 signaling pathway through a unique mechanism in GBM development.

### 7.9 zDHHC18 and zDHHC23

Bmi1 belongs to the Polycomb group (PcG) and is a key marker of stem cells (Xu et al., 2022). Initially discovered as an oncogene in mouse lymphomas, Bmi1 was later identified as an important regulator of hematopoietic and neural stem cell self-renewal (Wang et al., 2007). In this context, the role of zDHHC23 becomes evident as it recruits BMI1 and RNF144A (an E3 ligase involved in post-translational regulation of BMI1) to increase the ubiquitination level of BMI1 in susceptible neural stem cells, ensuring the stability of BMI1 protein in different subtypes of GSCs (Chen et al., 2019). On the other hand, ZDHHC18 affects the binding of BMI1 and RNF144A and reduces the polyubiquitination level of BMI1 in mesenchymal neural stem cells through competitive interaction with RNF144A. Overall, the relative changes in the abundance of zDHHC18 and zDHHC23 can regulate the expression pattern of BMI1, playing an important promoting role in the transition of glioblastoma stem cell states (Chen et al., 2019). These findings provide a new perspective for understanding the pathogenesis of GBM and lay the theoretical foundation for developing therapeutic strategies.

### 7.10 zDHHC19

**FIGURE 3:**
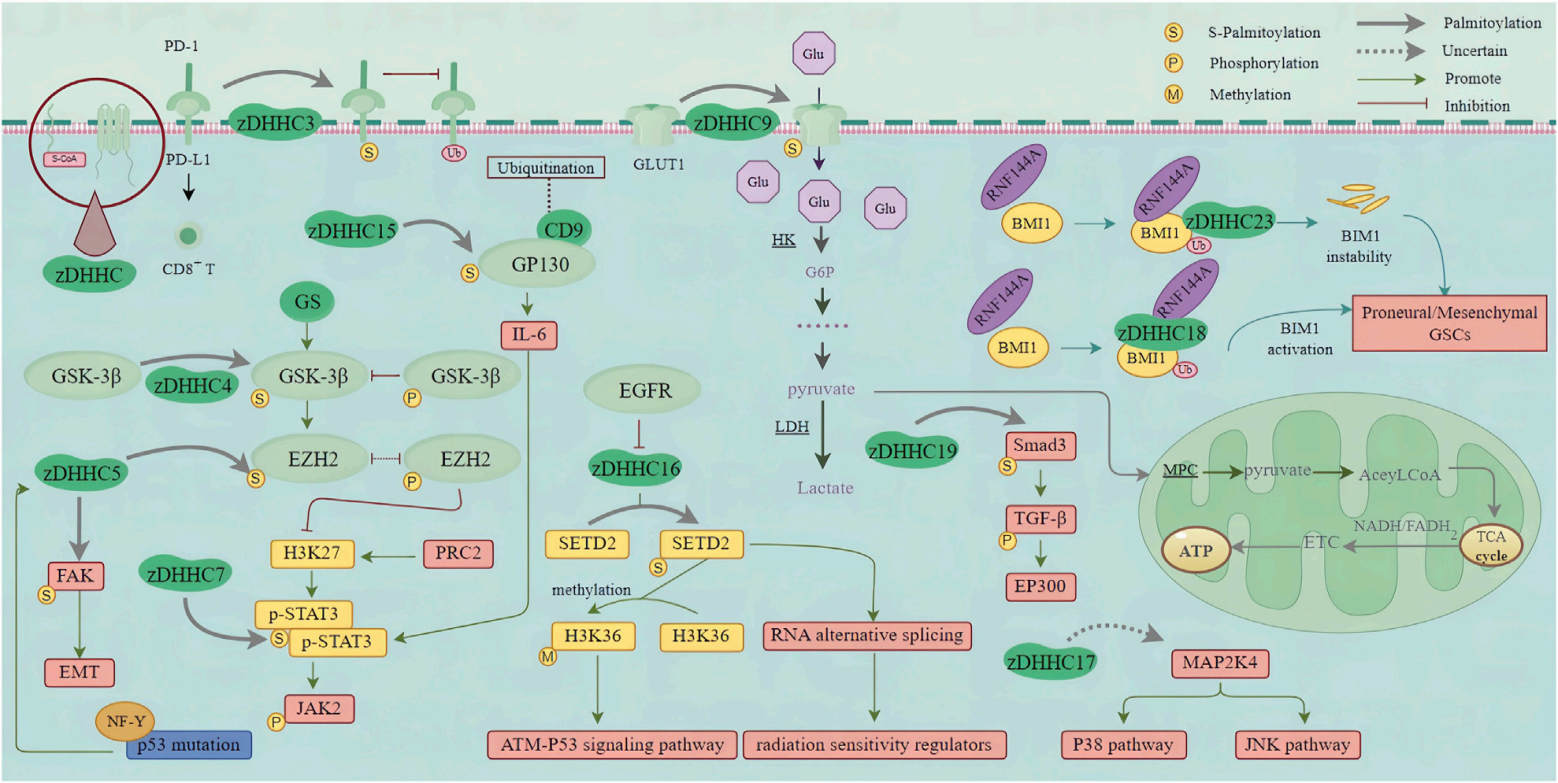
zDHHC-mediated S-palmitoylation in GBM Oncogenic Pathways. This figure illustrates the role of zDHHC enzymes in the S-palmitoylation of proteins involved in major oncogenic pathways in glioblastoma multiforme (GBM). zDHHC enzymes catalyze the addition of palmitoyl groups to specific proteins, such as EGFR, HRAS, and so on, which are critical in GBM pathogenesis. S-palmitoylation of these proteins affects their localization, stability, and interaction with other signaling molecules, thereby modulating key signaling pathways that promote GBM progression and malignancy. (PD-1, Programmed death receptor 1; PD-L1, Programmed death ligand 1; GSK-3β, Glycogen synthase kinase-3β; EZH2, Enhancer of Zeste homolog 2; GS, Glycogen synthase; FAK, Focal adhesion kinase; EMT, Epithelial-Mesenchymal Transition; NF-Y, Nuclear factor Y; H3K27, Histone 3 Lys 27; PRC2, Polycomb Repressive Complex 2; STAT3, Signal transducer and activator of transcription 3; JAK2, Janus kinase-2; CD9, Cluster of differentiation 9; GP130, Glycoprotein 130; IL-6, Interleukin 6; Glu, Glucose; GLUT1, Glucose transporter; HK, Hexokinase; G6P, Glucose 6-phosphate; LDH, lactate dehydrogenase; MPC, Mitochondrial pyruvate carrier; TCA cycle, Tricarboxylic acid cycle; ETC., Electron transfer chain; ATP, Adenosine triphosphate; Smad3, Small mother against decapentaplegic 3; TGF-β, Transforming growth factor-β; EP300, E1A-binding protein P300; MAP2K4, Mitogen-activated protein kinase kinase 4; JNK pathway, C-jun N-terminal kinase pathway; EGFR, Epidermal growth factor receptor; SETD2, Set2 in yeast; H3K36, Histone H3 on lysine 36; ATM, Ataxia Telangiectasia Mutated; RNA, Ribonucleic Acid; RNF144A, Ring Finger Protein 144; BMI1, B cell-specific Moloney murine leukemia virus integration site 1; GSCs, Glioma stem cells).

Due to the significant intra- and inter-tumoral heterogeneity of GBM (including cellular and molecular complexity, therapies targeting a single molecular pathway are ineffective for GBM (Balça-Silva et al., 2019; Lah et al., 2020). GBM contains glioblastoma stem cells (GSCs), which are highly resistant to radiotherapy and chemotherapy, leading to high recurrence rates (Bao et al., 2006; Sharifzad et al., 2019). Targeting GSCs and identifying new markers are crucial issues in developing innovative strategies to eradicate GBM. IDH1 mutation induces the activation of HIF-1α and reduces the expression of TGF-β1 in proneural GSCs. However, a mechanism has been identified in mesenchymal-type GSCs whereby Smad3 activation is controlled through the regulation of Smad3 palmitoylation. This palmitoylation, facilitated by membrane localization and TGF-β1/2 phosphorylation, promotes Smad3 activation (Fan et al., 2021b). Inhibiting the activity of HIF-1α and Smad3 may effectively suppress the proneural and mesenchymal subtypes of GBM. EP300, a histone acetyltransferase (HAT), is involved in the formation of transcription complexes and plays crucial roles in cell cycle regulation and DNA damage repair (Snowden and Perkins, 1998). Interaction between activated TGF-β and EP300 further enhances the expression of corresponding markers in mesenchymal-type GBM (Fan et al., 2021b). This discovery reveals the intricate regulatory relationship between IDH1 mutation, the TGF-β signaling pathway, and mesenchymal features (Figure 3).

### 7.11 Other zDHHC molecules

zDHHC2 is an important gene associated with GBM. Studies have identified zDHHC2 as a significant prognostic gene for GBM, closely linked to its prognosis (Yang H. et al., 2019). High expression of zDHHC2 in GBM is strongly associated with the “surfactant metabolism” pathway and has been validated as a prognostic marker for GBM. Additionally, zDHHC2 has been found to be prognostically relevant in various cancers. For instance, in gastric cancer, its downregulation is associated with lymph node metastasis and poor prognosis (Yan et al., 2013). While the exact mechanisms of zDHHC2 in GBM remain incompletely understood, research suggests that zDHHC2 may play a pivotal role in the pathogenesis and prognosis of GBM.

Previous studies have linked zDHHC12 to neurological disorders such as Huntington’s disease, Alzheimer’s disease, and schizophrenia (Liao et al., 2024; Young et al., 2012). Knocking out zDHHC12 in ovarian cancer significantly inhibits the precise membrane localization and protein stability of CLDN3, as well as tumor occurrence in ovarian cancer cells (Yuan et al., 2020b). Lu et al. (Lu et al., 2022) demonstrated the significant role of zDHHC12 in GBM. Firstly, their comprehensive analysis revealed upregulation of ZDHHC12 in various cancers, predicting poor prognosis in GBM and low-grade glioma (LGG). Further cell experiments indicated that zDHHC12 promotes the occurrence and progression of gliomas. Moreover, the study found that zDHHC12 exhibits a hypomethylated state in GBM, potentially being a mechanism underlying its overexpression. Hypomethylation of the zDHHC12 gene is particularly significant in high-grade glioma samples and is associated with IDH wild-type samples, suggesting that the methylation status of the zDHHC12 gene may occur before the increase in zDHHC12 expression, providing a potential biomarker for early screening of gliomas. While *in vitro* cell experiments demonstrate the role of zDHHC12 in the occurrence and progression of gliomas, further research is needed to collect more glioma samples and relevant clinical data to assess the performance of zDHHC12 in glioma diagnosis and prognosis.

## 8 Advances and strategies for targeting S-palmitoylation with drugs

Currently, research on the translational aspects of S-palmitoylation is progressing relatively slowly compared to other lipidation studies. However, selectively regulating one or more of the dozens of proteins involved in palmitoylation offers a broad and promising opportunity for the future. This regulation could potentially leverage the dynamic palmitoylation cycle to upregulate or downregulate the activity of palmitoylated tumor proteins or tumor suppressors that are otherwise difficult to target with drugs. The plasticity of palmitoylation indicates that a deeper understanding of its regulatory mechanisms could aid in the discovery of novel therapeutics.

Identifying inhibitors for several ZDHHCs or APTs is similar to developing “selective hybrid” kinase inhibitors, which already have recognized therapeutic value (Morphy, 2010). Although membrane-proximal cysteine is a common modification site, the lack of a consensus sequence for palmitoylation poses significant challenges for analysis. This variable and hydrophobic PTM makes it difficult to quantify the true extent and stoichiometry of palmitoylation. Therefore, new technological innovations may be required to enable knowledge-guided selection of specific ZDHHCs for drug discovery. For instance, recently reported chemical genetics methods have linked the metabolic labeling of palmitoylation with the activity of individual ZDHHCs, identifying specific relationships between ZDHHCs and their substrates (Ocasio et al., 2024; Tomić et al., 2024; Puthenveetil et al., 2023).

The ZDHHC proteins have a conserved hydrophobic lipid-binding site, indicating that these proteins can bind to small molecule targets (Rana et al., 2018). Although there are currently no fully validated, highly selective ZDHHC inhibitors, some APT inhibitors have demonstrated varying degrees of efficacy and selectivity (Won et al., 2016; Remsberg et al., 2021; Lan et al., 2021). Unfortunately, recent studies have tended to misuse nonspecific active compounds, such as 2-BP or cerulenin, as so-called ZDHHC inhibitors. These compounds primarily target lipid biosynthetic enzymes and have highly nonspecific and pleiotropic effects on ZDHHC activity (Lanyon-Hogg et al., 2017; Ergun et al., 2019; Davda et al., 2013).

The recent high-throughput screening for ZDHHC2 inhibitors represents the first step towards developing more selective ZDHHC inhibitors. However, the disclosed compounds were initially reported as inhibitors for another class of enzymes, so their selectivity needs further optimization (Salaun et al., 2022). For example, ZDHHC9 maintains the palmitoylation and plasma membrane expression of GLUT1, which is crucial for glucose transport in glioblastoma cells. Additionally, ZDHHC9 enhances anticancer immune suppression by catalyzing the palmitoylation of PD1 and PDL1 (67).

Directly targeting palmitoylation sites is an attractive alternative strategy due to its potential for high specificity. However, in practice, this approach may be limited to a few sites with well-defined binding pockets that are relatively buried, similar to TEAD inhibitors (Hagenbeek et al., 2023).

## 9 Conclusion and future perspectives

Glioblastoma (GBM) is a highly recurrent primary malignant tumor of the brain, which has long been a focus of clinical and research attention. In the studies of GBM, S-palmitoylation modification, as an important protein lipid modification, plays a crucial role in the occurrence, development, and treatment of tumors. This review summarizes the biological processes of S-palmitoylation modification and its key roles in GBM. Specifically, we focus on the zDHHC family as key enzymes of S-palmitoylation modification, their research progress, and potential roles in GBM.

Firstly, S-palmitoylation modification affects various cellular functions and signaling pathways by increasing the hydrophobicity and altering the localization of proteins. This modification plays a vital role in cell cycle regulation, cell proliferation, metastasis, and signal transduction, and its abnormal expression in tumors may lead to tumor initiation and progression.

Secondly, the zDHHC family, as crucial enzymes of S-palmitoylation modification, has attracted widespread attention in GBM research. Members of the zDHHC family may play different roles in the occurrence and development of GBM. We summarize the functions of molecules such as zDHHC3, zDHHC4, zDHHC5, zDHHC7, zDHHC9, zDHHC15, zDHHC16, zDHHC17, zDHHC18, and zDHHC23 in GBM and the signaling pathways they regulate. These molecules participate in the occurrence and progression of GBM by affecting tumor cell immune evasion, stem cell self-renewal, and activation of signaling pathways. We particularly highlight the regulation of key proteins’ palmitoylation modification by these molecules and their effects on tumor biological behaviors. For example, palmitoylation modification of PD-L1 mediated by zDHHC3 affects tumor cell immune evasion; palmitoylation modification of GSK-3β mediated by zDHHC4 is involved in glioblastoma stem cell self-renewal and resistance to chemotherapy drugs; palmitoylation modification of EZH2 mediated by zDHHC5 affects the self-renewal and metastasis ability of tumor stem cells.

With further research on the mechanisms of action of the zDHHC family in GBM, we can gain a deeper understanding of the roles of these molecules in tumor initiation and progression, providing new insights and targets for developing therapeutic strategies for GBM. For example, inhibiting tumor cell growth and metastasis by interfering with palmitoylation modification mediated by zDHHC molecules may become one of the future treatment strategies. Furthermore, exploring the relationship between the expression levels of these molecules in tumor tissues and clinical features can explore their potential value as diagnostic and prognostic markers.

However, S-palmitoylation faces some challenges and difficulties in GBM research. Firstly, the complexity of this field requires interdisciplinary cooperation, integrating expertise from biology, chemistry, bioinformatics, and other fields. Secondly, as a membrane modification process, although some progress has been made in research, a comprehensive understanding of the precise mechanism of S-palmitoylation in cells involves many complex biochemical processes and interactions, including specific steps of protein modification, enzymes involved, signaling pathways, etc. Current scientific research still needs to delve deeper to reveal the detailed mechanism of S-palmitoylation. Additionally, the heterogeneity and highly malignant characteristics of GBM itself add to the complexity of research, requiring more refined experimental design and data analysis.

Given the importance of S-palmitoylation mediated by the zDHHC family in GBM, future research can focus on several aspects: firstly, in-depth exploration of the expression patterns of each zDHHC molecule in GBM and their association with tumor development; secondly, studying the mechanisms by which each zDHHC molecule affects biological behaviors such as proliferation, invasion, and metastasis of GBM cells; finally, exploring the potential role of regulating or inhibiting the zDHHC family in GBM treatment, aiming to provide a theoretical basis and clinical application prospects for developing new therapeutic strategies.

Overall, the study of S-palmitoylation in GBM has broad prospects and important clinical application value. By overcoming the corresponding challenges, we hope to provide more effective and personalized treatment strategies for GBM patients, promoting the development of the tumor research field.

## References

[B1] Abrami L. Dallavilla T. Sandoz P. A. Demir M. Kunz B. Savoglidis G. (2017). Identification and dynamics of the human ZDHHC16-ZDHHC6 palmitoylation cascade. eLife 6, e27826. 10.7554/eLife.27826 28826475 PMC5582869

[B2] Anido J. Sáez-Borderías A. Gonzàlez-Juncà A. Rodón L. Folch G. Carmona M. A. (2010). TGF-Β receptor inhibitors target the CD44(high)/id1(high) glioma-initiating cell population in human glioblastoma. Cancer Cell 18, 655–668. 10.1016/j.ccr.2010.10.023 21156287

[B3] Ashenhurst J. (2015). Thiols and thioethers. Master Org. Chem. Available at: https://www.masterorganicchemistry.com/2015/07/05/thiols-and-thioethers/ (Accessed July 5, 2024).

[B4] Balça-Silva J. Matias D. Carmo A. do Sarmento-Ribeiro A. B. Lopes M. C. Moura-Neto V. (2019). Cellular and molecular mechanisms of glioblastoma malignancy: implications in resistance and therapeutic strategies. Semin. Cancer Biol. 58, 130–141. 10.1016/j.semcancer.2018.09.007 30266571

[B5] Bao S. Wu Q. McLendon R. E. Hao Y. Shi Q. Hjelmeland A. B. (2006). Glioma stem cells promote radioresistance by preferential activation of the DNA damage response. Nature 444, 756–760. 10.1038/nature05236 17051156

[B6] Becker J. Wilting J. (2019). WNT signaling in neuroblastoma. Cancers 11, 1013. 10.3390/cancers11071013 31331081 PMC6679057

[B7] Bharadwaj M. Bizzozero O. A. (1995). Myelin P0 glycoprotein and a synthetic peptide containing the palmitoylation site are both autoacylated. J. Neurochem. 65, 1805–1815. 10.1046/j.1471-4159.1995.65041805.x 7561879

[B8] Blanc M. David F. Abrami L. Migliozzi D. Armand F. Bürgi J. (2015). SwissPalm: protein palmitoylation database. F1000Research 4, 261. 10.12688/f1000research.6464.1 26339475 PMC4544385

[B9] Blaskovic S. Blanc M. van der Goot F. G. (2013). What does S-palmitoylation do to membrane proteins? FEBS J. 280, 2766–2774. 10.1111/febs.12263 23551889

[B10] Bollu L. R. Katreddy R. R. Blessing A. M. Pham N. Zheng B. Wu X. (2015). Intracellular activation of EGFR by fatty acid synthase dependent palmitoylation. Oncotarget 6, 34992–35003. 10.18632/oncotarget.5252 26378037 PMC4741504

[B11] Brennan C. W. Verhaak R. G. W. McKenna A. Campos B. Noushmehr H. Salama S. R. (2013). The somatic genomic landscape of glioblastoma. Cell 155, 462–477. 10.1016/j.cell.2013.09.034 24120142 PMC3910500

[B12] Burnaevskiy N. Peng T. Reddick L. E. Hang H. C. Alto N. M. (2015). Myristoylome profiling reveals a concerted mechanism of ARF GTPase deacylation by the bacterial protease IpaJ. Mol. Cell 58, 110–122. 10.1016/j.molcel.2015.01.040 25773595 PMC4385471

[B13] Busquets-Hernández C. Triola G. (2021). Palmitoylation as a key regulator of ras localization and function. Front. Mol. Biosci. 8, 659861. 10.3389/fmolb.2021.659861 33816563 PMC8010249

[B14] Carro M. S. Lim W. K. Alvarez M. J. Bollo R. J. Zhao X. Snyder E. Y. (2010). The transcriptional network for mesenchymal transformation of brain tumours. Nature 463, 318–325. 10.1038/nature08712 20032975 PMC4011561

[B15] Carta G. Murru E. Banni S. Manca C. (2017). Palmitic acid: physiological role, metabolism and nutritional implications. Front. Physiol. 8, 902. 10.3389/fphys.2017.00902 29167646 PMC5682332

[B16] Carta G. Murru E. Lisai S. Sirigu A. Piras A. Collu M. (2015). Dietary triacylglycerols with palmitic acid in the sn-2 position modulate levels of N-acylethanolamides in rat tissues. PloS One 10, e0120424. 10.1371/journal.pone.0120424 25775474 PMC4361611

[B17] Chamberlain L. H. Shipston M. J. (2015). The physiology of protein S-acylation. Physiol. Rev. 95, 341–376. 10.1152/physrev.00032.2014 25834228 PMC4551212

[B18] Chan P. Han X. Zheng B. DeRan M. Yu J. Jarugumilli G. K. (2016). Autopalmitoylation of TEAD proteins regulates transcriptional output of the Hippo pathway. Nat. Chem. Biol. 12, 282–289. 10.1038/nchembio.2036 26900866 PMC4798901

[B19] Chang G. Xie G. S. Ma L. Li P. Li L. Richard H. T. (2023). USP36 promotes tumorigenesis and drug sensitivity of glioblastoma by deubiquitinating and stabilizing ALKBH5. Neuro-Oncol 25, 841–853. 10.1093/neuonc/noac238 36239338 PMC10158114

[B20] Charollais J. Van Der Goot F. G. (2009). Palmitoylation of membrane proteins (Review). Mol. Membr. Biol. 26, 55–66. 10.1080/09687680802620369 19085289

[B21] Chen B. Sun Y. Niu J. Jarugumilli G. K. Wu X. (2018). Protein lipidation in cell signaling and diseases: function, regulation, and therapeutic opportunities. Cell Chem. Biol. 25, 817–831. 10.1016/j.chembiol.2018.05.003 29861273 PMC6054547

[B22] Chen J. J. Fan Y. Boehning D. (2021). Regulation of dynamic protein S-acylation. Front. Mol. Biosci. 8, 656440. 10.3389/fmolb.2021.656440 33981723 PMC8107437

[B23] Chen M.-H. Li Y.-J. Kawakami T. Xu S.-M. Chuang P.-T. (2004). Palmitoylation is required for the production of a soluble multimeric Hedgehog protein complex and long-range signaling in vertebrates. Genes Dev. 18, 641–659. 10.1101/gad.1185804 15075292 PMC387240

[B24] Chen S. Zhu B. Yin C. Liu W. Han C. Chen B. (2017a). Palmitoylation-dependent activation of MC1R prevents melanomagenesis. Nature 549, 399–403. 10.1038/nature23887 28869973 PMC5902815

[B25] Chen X. Hao A. Li X. Ye K. Zhao C. Yang H. (2020b). Activation of JNK and p38 MAPK mediated by ZDHHC17 drives glioblastoma multiforme development and malignant progression. Theranostics 10, 998–1015. 10.7150/thno.40076 31938047 PMC6956818

[B26] Chen X. Hu L. Yang H. Ma H. Ye K. Zhao C. (2019). DHHC protein family targets different subsets of glioma stem cells in specific niches. J. Exp. Clin. Cancer Res. CR 38, 25. 10.1186/s13046-019-1033-2 30658672 PMC6339410

[B27] Chen X. Li H. Fan X. Zhao C. Ye K. Zhao Z. (2020a). Protein palmitoylation regulates cell survival by modulating XBP1 activity in glioblastoma multiforme. Mol. Ther. Oncolytics 17, 518–530. 10.1016/j.omto.2020.05.007 33024813 PMC7525067

[B28] Chen X. Ma H. Wang Z. Zhang S. Yang H. Fang Z. (2017b). EZH2 palmitoylation mediated by ZDHHC5 in p53-mutant glioma drives malignant development and progression. Cancer Res. 77, 4998–5010. 10.1158/0008-5472.CAN-17-1139 28775165

[B29] Chen Z. Q. Ulsh L. S. DuBois G. Shih T. Y. (1985). Posttranslational processing of p21 ras proteins involves palmitylation of the C-terminal tetrapeptide containing cysteine-186. J. Virol. 56, 607–612. 10.1128/JVI.56.2.607-612.1985 2997480 PMC252618

[B30] Chong X. Zhu L. Yu D. Chen S. Wang G. Yu Q. (2023). ZDHHC9 promotes colon tumor growth by inhibiting effector T cells. Oncol. Lett. 25, 5. 10.3892/ol.2022.13591 36419754 PMC9677519

[B31] Cron L. Allen T. Febbraio M. A. (2016). The role of gp130 receptor cytokines in the regulation of metabolic homeostasis. J. Exp. Biol. 219, 259–265. 10.1242/jeb.129213 26792338

[B32] C Z. H Y. X F. W N. J F. S S. (2022). GSK3β palmitoylation mediated by ZDHHC4 promotes tumorigenicity of glioblastoma stem cells in temozolomide-resistant glioblastoma through the EZH2-STAT3 axis. Oncogenesis, 11. 10.1038/s41389-022-00402-w 35606353 PMC9126914

[B33] Daniel P. Meehan B. Sabri S. Jamali F. Sarkaria J. N. Choi D. (2022). Detection of temozolomide-induced hypermutation and response to PD-1 checkpoint inhibitor in recurrent glioblastoma. Neurooncol Adv. 4, vdac076. 10.1093/noajnl/vdac076 35795471 PMC9252128

[B34] Darmanis S. Sloan S. A. Croote D. Mignardi M. Chernikova S. Samghababi P. (2017). Single-cell RNA-seq analysis of infiltrating neoplastic cells at the migrating front of human glioblastoma. Cell Rep. 21, 1399–1410. 10.1016/j.celrep.2017.10.030 29091775 PMC5810554

[B35] Da Ros M. De Gregorio V. Iorio A. L. Giunti L. Guidi M. De Martino M. (2018). Glioblastoma chemoresistance: the double play by microenvironment and blood-brain barrier. Int. J. Mol. Sci. 19, 2879. 10.3390/ijms19102879 30248992 PMC6213072

[B36] Das T. Yount J. S. Hang H. C. (2021). Protein S-palmitoylation in immunity. Open Biol. 11, 200411. 10.1098/rsob.200411 33653086 PMC8061762

[B37] Davda D. El Azzouny M. A. Tom CTMB Hernandez J. L. Majmudar J. D. Kennedy R. T. (2013). Profiling targets of the irreversible palmitoylation inhibitor 2-bromopalmitate. ACS Chem. Biol. 8, 1912–1917. 10.1021/cb400380s 23844586 PMC3892994

[B38] Ergun S. L. Fernandez D. Weiss T. M. Li L. (2019). STING polymer structure reveals mechanisms for activation, hyperactivation, and inhibition. Cell 178, 290–301. 10.1016/j.cell.2019.05.036 31230712

[B39] Erices J. I. Bizama C. Niechi I. Uribe D. Rosales A. Fabres K. (2023). Glioblastoma microenvironment and invasiveness: new insights and therapeutic targets. Int. J. Mol. Sci. 24, 7047. 10.3390/ijms24087047 37108208 PMC10139189

[B40] Fan X. Fan J. Yang H. Zhao C. Niu W. Fang Z. (2021b). Heterogeneity of subsets in glioblastoma mediated by Smad3 palmitoylation. Oncogenesis 10, 72. 10.1038/s41389-021-00361-8 34707087 PMC8551152

[B41] Fan X. Sun S. Yang H. Ma H. Zhao C. Niu W. (2022). SETD2 palmitoylation mediated by ZDHHC16 in epidermal growth factor receptor-mutated glioblastoma promotes ionizing radiation-induced DNA damage. Int. J. Radiat. Oncol. Biol. Phys. 113, 648–660. 10.1016/j.ijrobp.2022.02.018 35192890

[B42] Fan X. Yang H. Zhao C. Hu L. Wang D. Wang R. (2021a). Local anesthetics impair the growth and self-renewal of glioblastoma stem cells by inhibiting ZDHHC15-mediated GP130 palmitoylation. Stem Cell Res. Ther. 12, 107. 10.1186/s13287-021-02175-2 33541421 PMC7863430

[B43] Feng C. Zhang L. Chang X. Qin D. Zhang T. (2023). Regulation of post-translational modification of PD-L1 and advances in tumor immunotherapy. Front. Immunol. 14, 1230135. 10.3389/fimmu.2023.1230135 37554324 PMC10405826

[B44] Fhu C. W. Ali A. (2021). Protein lipidation by palmitoylation and myristoylation in cancer. Front. Cell Dev. Biol. 9, 673647. 10.3389/fcell.2021.673647 34095144 PMC8173174

[B45] Fredericks G. J. Hoffmann F. W. Rose A. H. Osterheld H. J. Hess F. M. Mercier F. (2014). Stable expression and function of the inositol 1,4,5-triphosphate receptor requires palmitoylation by a DHHC6/selenoprotein K complex. Proc. Natl. Acad. Sci. U. S. A. 111, 16478–16483. 10.1073/pnas.1417176111 25368151 PMC4246275

[B46] Freeman G. J. Long A. J. Iwai Y. Bourque K. Chernova T. Nishimura H. (2000). Engagement of the PD-1 immunoinhibitory receptor by a novel B7 family member leads to negative regulation of lymphocyte activation. J. Exp. Med. 192, 1027–1034. 10.1084/jem.192.7.1027 11015443 PMC2193311

[B47] Ganapathy V. Thangaraju M. Prasad P. D. (2009). Nutrient transporters in cancer: relevance to Warburg hypothesis and beyond. Pharmacol. Ther. 121, 29–40. 10.1016/j.pharmthera.2008.09.005 18992769

[B48] Gorleku O. A. Barns A.-M. Prescott G. R. Greaves J. Chamberlain L. H. (2011). Endoplasmic reticulum localization of DHHC palmitoyltransferases mediated by lysine-based sorting signals. J. Biol. Chem. 286, 39573–39584. 10.1074/jbc.M111.272369 21926431 PMC3234780

[B49] Gottlieb C. D. Zhang S. Linder M. E. (2015). The cysteine-rich domain of the DHHC3 palmitoyltransferase is palmitoylated and contains tightly bound zinc. J. Biol. Chem. 290, 29259–29269. 10.1074/jbc.M115.691147 26487721 PMC4705932

[B50] Greaves J. Chamberlain L. H. (2011). DHHC palmitoyl transferases: substrate interactions and (patho)physiology. Trends Biochem. Sci. 36, 245–253. 10.1016/j.tibs.2011.01.003 21388813

[B51] Hagenbeek T. J. Zbieg J. R. Hafner M. Mroue R. Lacap J. A. Sodir N. M. (2023). An allosteric pan-TEAD inhibitor blocks oncogenic YAP/TAZ signaling and overcomes KRAS G12C inhibitor resistance. Nat. Cancer 4, 812–828. 10.1038/s43018-023-00577-0 37277530 PMC10293011

[B52] Hanahan D. (2022). Hallmarks of cancer: new dimensions. Cancer Discov. 12, 31–46. 10.1158/2159-8290.CD-21-1059 35022204

[B53] He R. Du S. Lei T. Xie X. Wang Y. (2020). Glycogen synthase kinase 3β in tumorigenesis and oncotherapy (Review). Oncol. Rep. 44, 2373–2385. 10.3892/or.2020.7817 33125126 PMC7610307

[B54] Hg J. Gi B. Jd B. Mj B. Mj C. Yt C. (2002). Nomenclature of the GLUT/SLC2A family of sugar/polyol transport facilitators. Am. J. Physiol. Endocrinol. Metab., 282. 10.1152/ajpendo.00407.2001 11882521

[B55] Hornemann T. (2015). Palmitoylation and depalmitoylation defects. J. Inherit. Metab. Dis. 38, 179–186. 10.1007/s10545-014-9753-0 25091425

[B56] Huang T. Yang Y. Song X. Wan X. Wu B. Sastry N. (2021). PRMT6 methylation of RCC1 regulates mitosis, tumorigenicity, and radiation response of glioblastoma stem cells. Mol. Cell 81, 1276–1291.e9. 10.1016/j.molcel.2021.01.015 33539787 PMC7979509

[B57] Jansen M. Beaumelle B. (2022). How palmitoylation affects trafficking and signaling of membrane receptors. Biol. Cell 114, 61–72. 10.1111/boc.202100052 34738237

[B58] Jennings B. C. Linder M. E. (2012). DHHC protein S-acyltransferases use similar ping-pong kinetic mechanisms but display different acyl-CoA specificities. J. Biol. Chem. 287, 7236–7245. 10.1074/jbc.M111.337246 22247542 PMC3293542

[B59] Jiang H. Yu K. Li M. Cui Y. Ren X. Yang C. (2020). Classification of progression patterns in glioblastoma: analysis of predictive factors and clinical implications. Front. Oncol. 10, 590648. 10.3389/fonc.2020.590648 33251147 PMC7673412

[B60] Jiang H. Zhang X. Chen X. Aramsangtienchai P. Tong Z. Lin H. (2018). Protein lipidation: occurrence, mechanisms, biological functions, and enabling technologies. Chem. Rev. 118, 919–988. 10.1021/acs.chemrev.6b00750 29292991 PMC5985209

[B61] Jin J. Zhi X. Wang X. Meng D. (2021). Protein palmitoylation and its pathophysiological relevance. J. Cell Physiol. 236, 3220–3233. 10.1002/jcp.30122 33094504

[B62] Johnson B. E. Mazor T. Hong C. Barnes M. Aihara K. McLean C. Y. (2014). Mutational analysis reveals the origin and therapy-driven evolution of recurrent glioma. Science 343, 189–193. 10.1126/science.1239947 24336570 PMC3998672

[B63] Kersten S. (2001). Mechanisms of nutritional and hormonal regulation of lipogenesis. EMBO Rep. 2, 282–286. 10.1093/embo-reports/kve071 11306547 PMC1083868

[B64] Kim E. Kim M. Woo D.-H. Shin Y. Shin J. Chang N. (2013). Phosphorylation of EZH2 activates STAT3 signaling via STAT3 methylation and promotes tumorigenicity of glioblastoma stem-like cells. Cancer Cell 23, 839–852. 10.1016/j.ccr.2013.04.008 23684459 PMC4109796

[B65] Kinney N. Varghese R. T. Anandakrishnan R. Garner H. R. S. (2017). ZDHHC3 as a risk and mortality marker for breast cancer in african American women. Cancer Inf. 16, 1176935117746644. 10.1177/1176935117746644 PMC5734450 29276372

[B66] Kmiec D. Lista M. J. Ficarelli M. Swanson C. M. Neil S. J. D. (2021). S-farnesylation is essential for antiviral activity of the long ZAP isoform against RNA viruses with diverse replication strategies. PLoS Pathog. 17, e1009726. 10.1371/journal.ppat.1009726 34695163 PMC8568172

[B67] Ko P.-J. Dixon S. J. (2018). Protein palmitoylation and cancer. EMBO Rep. 19, e46666. 10.15252/embr.201846666 30232163 PMC6172454

[B68] Komaki S. Sugita Y. Furuta T. Yamada K. Moritsubo M. Abe H. (2019). Expression of GLUT1 in pseudopalisaded and perivascular tumor cells is an independent prognostic factor for patients with glioblastomas. J. Neuropathol. Exp. Neurol. 78, 389–397. 10.1093/jnen/nly124 30990881 PMC6467190

[B69] Lah T. T. Novak M. Breznik B. (2020). Brain malignancies: glioblastoma and brain metastases. Semin. Cancer Biol. 60, 262–273. 10.1016/j.semcancer.2019.10.010 31654711

[B70] Lan T. Delalande C. Dickinson B. C. (2021). Inhibitors of DHHC family proteins. Curr. Opin. Chem. Biol. 65, 118–125. 10.1016/j.cbpa.2021.07.002 34467875 PMC8671176

[B71] Lanyon-Hogg T. Faronato M. Serwa R. A. Tate E. W. (2017). Dynamic protein acylation: new substrates, mechanisms, and drug targets. Trends Biochem. Sci. 42, 566–581. 10.1016/j.tibs.2017.04.004 28602500

[B72] Lee C.-J. Stix R. Rana M. S. Shikwana F. Murphy R. E. Ghirlando R. (2022). Bivalent recognition of fatty acyl-CoA by a human integral membrane palmitoyltransferase. Proc. Natl. Acad. Sci. U. S. A. 119, e2022050119. 10.1073/pnas.2022050119 35140179 PMC8851515

[B73] Lee J.-W. Hur J. Kwon Y.-W. Chae C.-W. Choi J.-I. Hwang I. (2021). KAI1(CD82) is a key molecule to control angiogenesis and switch angiogenic milieu to quiescent state. J. Hematol. OncolJ Hematol. Oncol. (2021) 14, 148. 10.1186/s13045-021-01147-6 PMC8444549 34530889

[B74] Lemonidis K. Gorleku O. A. Sanchez-Perez M. C. Grefen C. Chamberlain L. H. (2014). The Golgi S-acylation machinery comprises zDHHC enzymes with major differences in substrate affinity and S-acylation activity. Mol. Biol. Cell 25, 3870–3883. 10.1091/mbc.E14-06-1169 25253725 PMC4244197

[B75] Lemonidis K. Salaun C. Kouskou M. Diez-Ardanuy C. Chamberlain L. H. Greaves J. (2017). Substrate selectivity in the zDHHC family of S-acyltransferases. Biochem. Soc. Trans. 45, 751–758. 10.1042/BST20160309 28620036

[B76] Lemonidis K. Sanchez-Perez M. C. Chamberlain L. H. (2015). Identification of a novel sequence motif recognized by the ankyrin repeat domain of zDHHC17/13 S-acyltransferases. J. Biol. Chem. 290, 21939–21950. 10.1074/jbc.M115.657668 26198635 PMC4571948

[B77] Leske H. Camenisch Gross U. Hofer S. Neidert M. C. Leske S. Weller M. (2023). MGMT methylation pattern of long-term and short-term survivors of glioblastoma reveals CpGs of the enhancer region to be of high prognostic value. Acta Neuropathol. Commun. 11, 139. 10.1186/s40478-023-01622-w 37641156 PMC10463744

[B78] Levental I. Lyman E. (2023). Regulation of membrane protein structure and function by their lipid nano-environment. Nat. Rev. Mol. Cell Biol. 24, 107–122. 10.1038/s41580-022-00524-4 36056103 PMC9892264

[B79] Li H. Song C. Zhang Y. Liu G. Mi H. Li Y. (2024). Transgelin promotes glioblastoma stem cell hypoxic responses and maintenance through p53 acetylation. Adv. Sci. Weinh Baden-Wurtt Ger. 11, e2305620. 10.1002/advs.202305620 PMC10870072 38087889

[B80] Li X. Shen L. Xu Z. Liu W. Li A. Xu J. (2022). Protein palmitoylation modification during viral infection and detection methods of palmitoylated proteins. Front. Cell Infect. Microbiol. 12, 821596. 10.3389/fcimb.2022.821596 35155279 PMC8829041

[B81] Li Y. Gao Z. Wang Y. Pang B. Zhang B. Hu R. (2023). Lysine methylation promotes NFAT5 activation and determines temozolomide efficacy in glioblastoma. Nat. Commun. 14, 4062. 10.1038/s41467-023-39845-z 37429858 PMC10333326

[B82] Liao D. Huang Y. Liu D. Zhang H. Shi X. Li X. (2024). The role of s-palmitoylation in neurological diseases: implication for zDHHC family. Front. Pharmacol. 14, 1342830. 10.3389/fphar.2023.1342830 38293675 PMC10824933

[B83] Li J. Tong L. Yi L. Liu P. Yang X. Wang X. (2019). Experimental study on ACT001 inhibiting the spheroidization ability and stemness maintenance of U87-MG glioma stem cells through the STAT3 signaling pathway. Chin. J. Neurosurg. 35, 1160–1166. 10.3760/cma.j.issn.1001-2346.2019.11.019

[B84] Lim M. Xia Y. Bettegowda C. Weller M. (2018). Current state of immunotherapy for glioblastoma. Nat. Rev. Clin. Oncol. 15, 422–442. 10.1038/s41571-018-0003-5 29643471

[B85] Lin D. T. S. Davis N. G. Conibear E. (2017). Targeting the Ras palmitoylation/depalmitoylation cycle in cancer. Biochem. Soc. Trans. 45, 913–921. 10.1042/BST20160303 28630138

[B86] Lin H. Su X. He B. (2012). Protein lysine acylation and cysteine succination by intermediates of energy metabolism. ACS Chem. Biol. 7, 947–960. 10.1021/cb3001793 22571489 PMC3376250

[B87] Linder M. E. Deschenes R. J. (2007). Palmitoylation: policing protein stability and traffic. Nat. Rev. Mol. Cell Biol. 8, 74–84. 10.1038/nrm2084 17183362

[B88] Linder M. E. Jennings B. C. (2013). Mechanism and function of DHHC S-acyltransferases. Biochem. Soc. Trans. 41, 29–34. 10.1042/BST20120328 23356254

[B89] Liu R. Lee J.-H. Li J. Yu R. Tan L. Xia Y. (2021). Choline kinase alpha 2 acts as a protein kinase to promote lipolysis of lipid droplets. Mol. Cell 81, 2722–2735.e9. 10.1016/j.molcel.2021.05.005 34077757

[B90] Liu X. Guo C. Leng T. Fan Z. Mai J. Chen J. (2023a). Differential regulation of H3K9/H3K14 acetylation by small molecules drives neuron-fate-induction of glioma cell. Cell Death Dis. 14, 142. 10.1038/s41419-023-05611-8 36805688 PMC9941105

[B91] Liu Z. Liu C. Xiao M. Han Y. Zhang S. Xu B. (2020). Bioinformatics analysis of the prognostic and biological significance of ZDHHC-protein acyltransferases in kidney renal clear cell carcinoma. Front. Oncol. 10, 565414. 10.3389/fonc.2020.565414 33364189 PMC7753182

[B92] Liu Z.-Y. Lan T. Tang F. He Y.-Z. Liu J.-S. Yang J.-Z. (2023b). ZDHHC15 promotes glioma malignancy and acts as a novel prognostic biomarker for patients with glioma. BMC Cancer 23, 420. 10.1186/s12885-023-10883-6 37161425 PMC10169355

[B93] Lothrop A. P. Torres M. P. Fuchs S. M. (2013). Deciphering post-translational modification codes. FEBS Lett. 587, 1247–1257. 10.1016/j.febslet.2013.01.047 23402885 PMC3888991

[B94] Louis D. N. Perry A. Reifenberger G. von Deimling A. Figarella-Branger D. Cavenee W. K. (2016). The 2016 World health organization classification of tumors of the central nervous system: a summary. Acta Neuropathol. Berl. 131, 803–820. 10.1007/s00401-016-1545-1 27157931

[B95] Louis D. N. Perry A. Wesseling P. Brat D. J. Cree I. A. Figarella-Branger D. (2021). The 2021 WHO classification of tumors of the central nervous system: a summary. Neuro-Oncol 23, 1231–1251. 10.1093/neuonc/noab106 34185076 PMC8328013

[B96] Lu F. Shen S.-H. Wu S. Zheng P. Lin K. Liao J. (2022). Hypomethylation-induced prognostic marker zinc finger DHHC-type palmitoyltransferase 12 contributes to glioblastoma progression. Ann. Transl. Med. 10, 334. 10.21037/atm-22-520 35434031 PMC9011314

[B97] Macheda M. L. Rogers S. Best J. D. (2005). Molecular and cellular regulation of glucose transporter (GLUT) proteins in cancer. J. Cell Physiol. 202, 654–662. 10.1002/jcp.20166 15389572

[B98] Malgapo M. I. P. Linder M. E. (2021). Substrate recruitment by zDHHC protein acyltransferases. Open Biol. 11, 210026. 10.1098/rsob.210026 33878949 PMC8059564

[B99] Martin B. R. Wang C. Adibekian A. Tully S. E. Cravatt B. F. (2011). Global profiling of dynamic protein palmitoylation. Nat. Methods 9, 84–89. 10.1038/nmeth.1769 22056678 PMC3248616

[B100] Mesquita F. S. Abrami L. Sergeeva O. Turelli P. Qing E. Kunz B. (2021). S-acylation controls SARS-CoV-2 membrane lipid organization and enhances infectivity. Dev. Cell 56, 2790–2807.e8. 10.1016/j.devcel.2021.09.016 34599882 PMC8486083

[B101] Mg V. H. Lc C. Cb T. (2009). Understanding the Warburg effect: the metabolic requirements of cell proliferation. Science, 324. 10.1126/science.1160809 PMC2849637 19460998

[B102] Mitchell D. A. Vasudevan A. Linder M. E. Deschenes R. J. (2006). Protein palmitoylation by a family of DHHC protein S-acyltransferases. J. Lipid Res. 47, 1118–1127. 10.1194/jlr.R600007-JLR200 16582420

[B103] Mizoguchi M. Betensky R. A. Batchelor T. T. Bernay D. C. Louis D. N. Nutt C. L. (2006). Activation of STAT3, MAPK, and AKT in malignant astrocytic gliomas: correlation with EGFR status, tumor grade, and survival. J. Neuropathol. Exp. Neurol. 65, 1181–1188. 10.1097/01.jnen.0000248549.14962.b2 17146292

[B104] Morphy R. (2010). Selectively nonselective kinase inhibition: striking the right balance. J. Med. Chem. 53, 1413–1437. 10.1021/jm901132v 20166671

[B105] Mueckler M. Caruso C. Baldwin S. A. Panico M. Blench I. Morris H. R. (1985). Sequence and structure of a human glucose transporter. Science 229, 941–945. 10.1126/science.3839598 3839598

[B106] Nakada M. Kita D. Watanabe T. Hayashi Y. Teng L. Pyko I. V. (2011). Aberrant signaling pathways in glioma. Cancers 3 (3), 3242–3278. 10.3390/cancers3033242 24212955 PMC3759196

[B107] Nogueira-Silva C. Piairo P. Carvalho-Dias E. Veiga C. Moura R. S. Correia-Pinto J. (2013). The role of glycoprotein 130 family of cytokines in fetal rat lung development. PloS One 8, e67607. 10.1371/journal.pone.0067607 23826327 PMC3691159

[B108] Ocasio C. A. Baggelaar M. P. Sipthorp J. Losada de la Lastra A. Tavares M. Volarić J. (2024). A palmitoyl transferase chemical-genetic system to map ZDHHC-specific S-acylation. Nat. Biotechnol. 42, 1548–1558. 10.1038/s41587-023-02030-0 38191663 PMC11471619

[B109] Ohno Y. Kihara A. Sano T. Igarashi Y. (2006). Intracellular localization and tissue-specific distribution of human and yeast DHHC cysteine-rich domain-containing proteins. Biochim. Biophys. Acta 1761, 474–483. 10.1016/j.bbalip.2006.03.010 16647879

[B110] Ostrom Q. T. Price M. Neff C. Cioffi G. Waite K. A. Kruchko C. (2022). CBTRUS statistical report: primary brain and other central nervous system tumors diagnosed in the United States in 2015-2019. Neuro-Oncol 24, v1–v95. 10.1093/neuonc/noac202 36196752 PMC9533228

[B111] Pan S. Chen R. (2022). Pathological implication of protein post-translational modifications in cancer. Mol. Asp. Med. 86, 101097. 10.1016/j.mam.2022.101097 PMC9378605 35400524

[B112] Pastushenko I. Blanpain C. (2019). EMT transition states during tumor progression and metastasis. Trends Cell Biol. 29, 212–226. 10.1016/j.tcb.2018.12.001 30594349

[B113] Pavlova N. N. Zhu J. Thompson C. B. (2022). The hallmarks of cancer metabolism: still emerging. Cell Metab. 34, 355–377. 10.1016/j.cmet.2022.01.007 35123658 PMC8891094

[B114] Pei X. Li K.-Y. Shen Y. Li J.-T. Lei M.-Z. Fang C.-Y. (2022). Palmitoylation of MDH2 by ZDHHC18 activates mitochondrial respiration and accelerates ovarian cancer growth. Sci. China Life Sci. 65, 2017–2030. 10.1007/s11427-021-2048-2 35366151

[B115] Pei Z. Xiao Y. Meng J. Hudmon A. Cummins T. R. (2016). Cardiac sodium channel palmitoylation regulates channel availability and myocyte excitability with implications for arrhythmia generation. Nat. Commun. 7, 12035. 10.1038/ncomms12035 27337590 PMC4931030

[B116] Plain F. Howie J. Kennedy J. Brown E. Shattock M. J. Fraser N. J. (2020). Control of protein palmitoylation by regulating substrate recruitment to a zDHHC-protein acyltransferase. Commun. Biol. 3, 411. 10.1038/s42003-020-01145-3 32737405 PMC7395175

[B117] Puthenveetil R. Auger S. A. Gómez-Navarro N. Rana M. S. Das R. Healy L. B. (2023). Orthogonal enzyme-substrate design strategy for discovery of human protein palmitoyltransferase substrates. J. Am. Chem. Soc. 145, 22287–22292. 10.1021/jacs.3c04359 37774000 PMC10591334

[B118] Qu M. Zhou X. Wang X. Li H. (2021). Lipid-induced S-palmitoylation as a vital regulator of cell signaling and disease development. Int. J. Biol. Sci. 17, 4223–4237. 10.7150/ijbs.64046 34803494 PMC8579454

[B119] Quail D. F. Joyce J. A. (2017). The microenvironmental landscape of brain tumors. Cancer Cell 31, 326–341. 10.1016/j.ccell.2017.02.009 28292436 PMC5424263

[B120] Ramos A. K. S. Caldas-Rosa E. C. C. Ferreira B. M. Versiani B. R. Moretti P. N. de Oliveira S. F. (2023). ZDHHC9 X-linked intellectual disability: clinical and molecular characterization. Am. J. Med. Genet. A 191, 599–604. 10.1002/ajmg.a.63052 36416207

[B121] Rana M. S. Kumar P. Lee C.-J. Verardi R. Rajashankar K. R. Banerjee A. (2018). Fatty acyl recognition and transfer by an integral membrane S-acyltransferase. Science 359, eaao6326. 10.1126/science.aao6326 29326245 PMC6317078

[B122] Rana M. S. Lee C.-J. Banerjee A. (2019). The molecular mechanism of DHHC protein acyltransferases. Biochem. Soc. Trans. 47, 157–167. 10.1042/BST20180429 30559274

[B123] Remsberg J. R. Suciu R. M. Zambetti N. A. Hanigan T. W. Firestone A. J. Inguva A. (2021). ABHD17 regulation of plasma membrane palmitoylation and N-Ras-dependent cancer growth. Nat. Chem. Biol. 17, 856–864. 10.1038/s41589-021-00785-8 33927411 PMC8900659

[B124] Resh M. D. (2006a). Palmitoylation of ligands, receptors, and intracellular signaling molecules. Sci. STKE Signal Transduct. Knowl. Environ. 2006, re14. 10.1126/stke.3592006re14 17077383

[B125] Resh M. D. (2006b). Trafficking and signaling by fatty-acylated and prenylated proteins. Nat. Chem. Biol. 2, 584–590. 10.1038/nchembio834 17051234

[B126] Resh M. D. (2013). Covalent lipid modifications of proteins. Curr. Biol. CB 23, R431–R435. 10.1016/j.cub.2013.04.024 23701681 PMC3712495

[B127] Robert C. Vagner S. (2018). Boosting immunity by targeting post-translational prenylation of small GTPases. Cell 175, 901–902. 10.1016/j.cell.2018.10.032 30388448

[B128] Rocks O. Peyker A. Kahms M. Verveer P. J. Koerner C. Lumbierres M. (2005). An acylation cycle regulates localization and activity of palmitoylated Ras isoforms. Science 307, 1746–1752. 10.1126/science.1105654 15705808

[B129] Rodenburg R. N. P. Snijder J. van de Waterbeemd M. Schouten A. Granneman J. Heck A. J. R. (2017). Stochastic palmitoylation of accessible cysteines in membrane proteins revealed by native mass spectrometry. Nat. Commun. 8, 1280. 10.1038/s41467-017-01461-z 29097667 PMC5668376

[B130] Roth A. F. Feng Y. Chen L. Davis N. G. (2002). The yeast DHHC cysteine-rich domain protein Akr1p is a palmitoyl transferase. J. Cell Biol. 159, 23–28. 10.1083/jcb.200206120 12370247 PMC2173492

[B131] Runkle K. B. Kharbanda A. Stypulkowski E. Cao X.-J. Wang W. Garcia B. A. (2016). Inhibition of DHHC20-mediated EGFR palmitoylation creates a dependence on EGFR signaling. Mol. Cell 62, 385–396. 10.1016/j.molcel.2016.04.003 27153536 PMC4860254

[B132] Salaun C. Locatelli C. Zmuda F. Cabrera González J. Chamberlain L. H. (2020). Accessory proteins of the zDHHC family of S-acylation enzymes. J. Cell Sci. 133, jcs251819. 10.1242/jcs.251819 33203738

[B133] Salaun C. Takizawa H. Galindo A. Munro K. R. McLellan J. Sugimoto I. (2022). Development of a novel high-throughput screen for the identification of new inhibitors of protein S-acylation. J. Biol. Chem. 298, 102469. 10.1016/j.jbc.2022.102469 36087837 PMC9558053

[B134] Schmidt M. F. Bracha M. Schlesinger M. J. (1979). Evidence for covalent attachment of fatty acids to Sindbis virus glycoproteins. Proc. Natl. Acad. Sci. U. S. A. 76, 1687–1691. 10.1073/pnas.76.4.1687 287008 PMC383455

[B135] Shahinian S. Silvius J. R. (1995). Doubly-lipid-modified protein sequence motifs exhibit long-lived anchorage to lipid bilayer membranes. Biochemistry 34, 3813–3822. 10.1021/bi00011a039 7893678

[B136] Sharifzad F. Ghavami S. Verdi J. Mardpour S. Mollapour Sisakht M. Azizi Z. (2019). Glioblastoma cancer stem cell biology: potential theranostic targets. Drug Resist Updat Rev. Comment Antimicrob. Anticancer Chemother. 42, 35–45. 10.1016/j.drup.2018.03.003 30877905

[B137] Shergalis A. Bankhead A. Luesakul U. Muangsin N. Neamati N. (2018). Current challenges and opportunities in treating glioblastoma. Pharmacol. Rev. 70, 412–445. 10.1124/pr.117.014944 29669750 PMC5907910

[B138] Shi Y. Zhou W. Cheng L. Chen C. Huang Z. Fang X. (2017). Tetraspanin CD9 stabilizes gp130 by preventing its ubiquitin-dependent lysosomal degradation to promote STAT3 activation in glioma stem cells. Cell Death Differ. 24, 167–180. 10.1038/cdd.2016.110 27740621 PMC5260495

[B139] Smotrys J. E. Linder M. E. (2004). Palmitoylation of intracellular signaling proteins: regulation and function. Annu. Rev. Biochem. 73, 559–587. 10.1146/annurev.biochem.73.011303.073954 15189153

[B140] Snowden A. W. Perkins N. D. (1998). Cell cycle regulation of the transcriptional coactivators p300 and CREB binding protein. Biochem. Pharmacol. 55, 1947–1954. 10.1016/S0006-2952(98)00020-3 9714314

[B141] Sobocińska J. Roszczenko-Jasińska P. Ciesielska A. Kwiatkowska K. (2018). Protein palmitoylation and its role in bacterial and viral infections. Front. Immunol. 8, 2003. 10.3389/fimmu.2017.02003 29403483 PMC5780409

[B142] Song Z. Xiaoli A. M. Yang F. (2018). Regulation and metabolic significance of *de novo* lipogenesis in adipose tissues. Nutrients 10, 1383. 10.3390/nu10101383 30274245 PMC6213738

[B143] Stix R. Lee C.-J. Faraldo-Gómez J. D. Banerjee A. (2020). Structure and mechanism of DHHC protein acyltransferases. J. Mol. Biol. 432, 4983–4998. 10.1016/j.jmb.2020.05.023 32522557 PMC7483407

[B144] Stommel J. M. Kimmelman A. C. Ying H. Nabioullin R. Ponugoti A. H. Wiedemeyer R. (2007). Coactivation of receptor tyrosine kinases affects the response of tumor cells to targeted therapies. Science 318, 287–290. 10.1126/science.1142946 17872411

[B145] Sun Y. Zhang H. Meng J. Guo F. Ren D. Wu H. (2022). S-palmitoylation of PCSK9 induces sorafenib resistance in liver cancer by activating the PI3K/AKT pathway. Cell Rep. 40, 111194. 10.1016/j.celrep.2022.111194 35977495

[B146] Sun Y. Zhu L. Liu P. Zhang H. Guo F. Jin X. (2023). ZDHHC2-Mediated AGK palmitoylation activates AKT-mTOR signaling to reduce sunitinib sensitivity in renal cell carcinoma. Cancer Res. 83, 2034–2051. 10.1158/0008-5472.CAN-22-3105 37078777 PMC10267682

[B147] Swarthout J. T. Lobo S. Farh L. Croke M. R. Greentree W. K. Deschenes R. J. (2005). DHHC9 and GCP16 constitute a human protein fatty acyltransferase with specificity for H- and N-Ras. J. Biol. Chem. 280, 31141–31148. 10.1074/jbc.M504113200 16000296

[B148] Tabaczar S. Czogalla A. Podkalicka J. Biernatowska A. Sikorski A. F. (2017). Protein palmitoylation: palmitoyltransferases and their specificity. Exp. Biol. Med. Maywood N. J. 242, 1150–1157. 10.1177/1535370217707732 PMC5478004 28485685

[B149] Tang F. Liu Z. Chen X. Yang J. Wang Z. Li Z. (2022a). Current knowledge of protein palmitoylation in gliomas. Mol. Biol. Rep. 49, 10949–10959. 10.1007/s11033-022-07809-z 36044113

[B150] Tang F. Yang C. Li F.-P. Yu D.-H. Pan Z.-Y. Wang Z.-F. (2022c). Palmitoyl transferases act as potential regulators of tumor-infiltrating immune cells and glioma progression. Mol. Ther. Nucleic Acids 28, 716–731. 10.1016/j.omtn.2022.04.030 35664705 PMC9126852

[B151] Tang W. Xu N. Zhou J. He Z. Lenahan C. Wang C. (2022b). ALKBH5 promotes PD-L1-mediated immune escape through m6A modification of ZDHHC3 in glioma. Cell Death Discov. 8, 497. 10.1038/s41420-022-01286-w 36566230 PMC9789960

[B152] Tate J. G. Bamford S. Jubb H. C. Sondka Z. Beare D. M. Bindal N. (2019). COSMIC: the catalogue of somatic mutations in cancer. Nucleic Acids Res. 47, D941-D947–D947. 10.1093/nar/gky1015 30371878 PMC6323903

[B153] T D. Iv P. T F. K M. M U. T S. (2016). Glycogen synthase kinase-3β is a pivotal mediator of cancer invasion and resistance to therapy. Cancer Sci., 107. 10.1111/cas.13028 27486911 PMC5084660

[B154] Thiery J. P. Acloque H. Huang R. Y. J. Nieto M. A. (2009). Epithelial-mesenchymal transitions in development and disease. Cell 139, 871–890. 10.1016/j.cell.2009.11.007 19945376

[B155] Tian H. Lu J.-Y. Shao C. Huffman K. E. Carstens R. M. Larsen J. E. (2015). Systematic siRNA screen unmasks NSCLC growth dependence by palmitoyltransferase DHHC5. Mol. Cancer Res. MCR 13, 784–794. 10.1158/1541-7786.MCR-14-0608 25573953 PMC4398612

[B156] Tomić G. Sheridan C. Refermat A. Y. Baggelaar M. P. Sipthorp J. Sudarshan B. (2024). Palmitoyl transferase ZDHHC20 promotes pancreatic cancer metastasis. Cell Rep. 43, 114224. 10.1016/j.celrep.2024.114224 38733589

[B157] Uldry M. Thorens B. (2004). The SLC2 family of facilitated hexose and polyol transporters. Pflüg Arch. 447, 480–489. 10.1007/s00424-003-1085-0 12750891

[B158] van Bergen L. A. H. Alonso M. Palló A. Nilsson L. De Proft F. Messens J. (2016). Revisiting sulfur H-bonds in proteins: the example of peroxiredoxin AhpE. Sci. Rep. 6, 30369. 10.1038/srep30369 27468924 PMC4965862

[B159] Verardi R. Kim J.-S. Ghirlando R. Banerjee A. (2017). Structural basis for substrate recognition by the ankyrin repeat domain of human DHHC17 palmitoyltransferase. Struct. Lond Engl. 25, 1337–1347. 10.1016/j.str.2017.06.018 PMC5599134 28757145

[B160] Verdugo E. Puerto I. Medina M. Á. (2022). An update on the molecular biology of glioblastoma, with clinical implications and progress in its treatment. Cancer Commun. Lond Engl. 42, 1083–1111. 10.1002/cac2.12361 PMC9648390 36129048

[B161] Wagener E.-M. Aurich M. Aparicio-Siegmund S. Floss D. M. Garbers C. Breusing K. (2014). The amino acid exchange R28E in ciliary neurotrophic factor (CNTF) abrogates interleukin-6 receptor-dependent but retains CNTF receptor-dependent signaling via glycoprotein 130 (gp130)/Leukemia inhibitory factor receptor (LIFR). J. Biol. Chem. 289, 18442–18450. 10.1074/jbc.M114.568857 24802752 PMC4140248

[B162] Wang G. G. Allis C. D. Chi P. (2007). Chromatin remodeling and cancer, part I: covalent histone modifications. Trends Mol. Med. 13, 363–372. 10.1016/j.molmed.2007.07.003 17822958

[B163] Wang L.-B. Karpova A. Gritsenko M. A. Kyle J. E. Cao S. Li Y. (2021). Proteogenomic and metabolomic characterization of human glioblastoma. Cancer Cell 39, 509–528.e20. 10.1016/j.ccell.2021.01.006 33577785 PMC8044053

[B164] Wang X. Zhang C. Bao N. (2023). Molecular mechanism of palmitic acid and its derivatives in tumor progression. Front. Oncol. 13, 1224125. 10.3389/fonc.2023.1224125 37637038 PMC10447256

[B165] Wang Y. Lu H. Fang C. Xu J. (2020). Palmitoylation as a signal for delivery. Adv. Exp. Med. Biol. 1248, 399–424. 10.1007/978-981-15-3266-5_16 32185719

[B166] Wang Y. Shen N. Yang Y. Xia Y. Zhang W. Lu Y. (2024). ZDHHC5-mediated S-palmitoylation of FAK promotes its membrane localization and epithelial-mesenchymal transition in glioma. Cell Commun. Signal CCS 22, 46. 10.1186/s12964-023-01366-z 38233791 PMC10795333

[B167] White K. Connor K. Clerkin J. Murphy B. M. Salvucci M. O’Farrell A. C. (2020). New hints towards a precision medicine strategy for IDH wild-type glioblastoma. Ann. Oncol. Off. J. Eur. Soc. Med. Oncol. 31, 1679–1692. 10.1016/j.annonc.2020.08.2336 32918998

[B168] Won S. J. Davda D. Labby K. J. Hwang S. Y. Pricer R. Majmudar J. D. (2016). Molecular mechanism for isoform-selective inhibition of acyl protein thioesterases 1 and 2 (APT1 and APT2). ACS Chem. Biol. 11, 3374–3382. 10.1021/acschembio.6b00720 27748579 PMC5359770

[B169] Won S. J. Martin B. R. (2018). Temporal profiling establishes a dynamic S-palmitoylation cycle. ACS Chem. Biol. 13, 1560–1568. 10.1021/acschembio.8b00157 29733200 PMC6192522

[B170] Xiao D. Yan C. Li D. Xi T. Liu X. Zhu D. (2023). National Brain Tumour Registry of China (NBTRC) statistical report of primary brain tumours diagnosed in China in years 2019-2020. Lancet Reg. Health West Pac 34, 100715. 10.1016/j.lanwpc.2023.100715 37283963 PMC10240383

[B171] Xu J. Li L. Shi P. Cui H. Yang L. (2022). The crucial roles of bmi-1 in cancer: implications in pathogenesis, metastasis, drug resistance, and targeted therapies. Int. J. Mol. Sci. 23, 8231. 10.3390/ijms23158231 35897796 PMC9367737

[B172] Yan S.-M. Tang J.-J. Huang C.-Y. Xi S.-Y. Huang M.-Y. Liang J.-Z. (2013). Reduced expression of ZDHHC2 is associated with lymph node metastasis and poor prognosis in gastric adenocarcinoma. PloS One 8, e56366. 10.1371/journal.pone.0056366 23457560 PMC3574152

[B173] Yang H. Jin L. Sun X. (2019b). A thirteen-gene set efficiently predicts the prognosis of glioblastoma. Mol. Med. Rep. 19, 1613–1621. 10.3892/mmr.2019.9801 30628650 PMC6390043

[B174] Yang Y. Hsu J.-M. Sun L. Chan L.-C. Li C.-W. Hsu J. L. (2019a). Palmitoylation stabilizes PD-L1 to promote breast tumor growth. Cell Res. 29, 83–86. 10.1038/s41422-018-0124-5 30514902 PMC6318320

[B175] Yao H. Lan J. Li C. Shi H. Brosseau J.-P. Wang H. (2019). Inhibiting PD-L1 palmitoylation enhances T-cell immune responses against tumours. Nat. Biomed. Eng. 3, 306–317. 10.1038/s41551-019-0375-6 30952982

[B176] Yao H. Li C. He F. Song T. Brosseau J.-P. Wang H. (2021). A peptidic inhibitor for PD-1 palmitoylation targets its expression and functions. RSC Chem. Biol. 2, 192–205. 10.1039/d0cb00157k 34458782 PMC8341464

[B177] Young F. B. Butland S. L. Sanders S. S. Sutton L. M. Hayden M. R. (2012). Putting proteins in their place: palmitoylation in Huntington disease and other neuropsychiatric diseases. Prog. Neurobiol. 97, 220–238. 10.1016/j.pneurobio.2011.11.002 22155432

[B178] Yuan M. Chen X. Sun Y. Jiang L. Xia Z. Ye K. (2020b). ZDHHC12-mediated claudin-3 S-palmitoylation determines ovarian cancer progression. Acta Pharm. Sin. B 10, 1426–1439. 10.1016/j.apsb.2020.03.008 32963941 PMC7488353

[B179] Yuan M. Song Z.-H. Ying M.-D. Zhu H. He Q.-J. Yang B. (2020a). N-myristoylation: from cell biology to translational medicine. Acta Pharmacol. Sin. 41, 1005–1015. 10.1038/s41401-020-0388-4 32203082 PMC7468318

[B180] Yuan Y. Li P. Li J. Zhao Q. Chang Y. He X. (2024). Protein lipidation in health and disease: molecular basis, physiological function and pathological implication. Signal Transduct. Target Ther. 9, 60. 10.1038/s41392-024-01759-7 38485938 PMC10940682

[B181] Zhang J. Huang Y. Chen J. Zhu H. Whiteheart S. W. (2018). Dynamic cycling of t-SNARE acylation regulates platelet exocytosis. J. Biol. Chem. 293, 3593–3606. 10.1074/jbc.RA117.000140 29352103 PMC5846156

[B182] Zhang M. Zhou L. Xu Y. Yang M. Xu Y. Komaniecki G. P. (2020). A STAT3 palmitoylation cycle promotes TH17 differentiation and colitis. Nature 586, 434–439. 10.1038/s41586-020-2799-2 33029007 PMC7874492

[B183] Zhang M. M. Hang H. C. (2017). Protein S-palmitoylation in cellular differentiation. Biochem. Soc. Trans. 45, 275–285. 10.1042/BST20160236 28202682 PMC5310721

[B184] Zhang Y. Qin Z. Sun W. Chu F. Zhou F. (2021a). Function of protein S-palmitoylation in immunity and immune-related diseases. Front. Immunol. 12, 661202. 10.3389/fimmu.2021.661202 34557182 PMC8453015

[B185] Zhang Z. Li X. Yang F. Chen C. Liu P. Ren Y. (2021b). DHHC9-mediated GLUT1 S-palmitoylation promotes glioblastoma glycolysis and tumorigenesis. Nat. Commun. 12, 5872. 10.1038/s41467-021-26180-4 34620861 PMC8497546

[B186] Zhong B. Yu J. Hou Y. Ai N. Ge W. Lu J. J. (2021). A novel strategy for glioblastoma treatment by induction of noptosis, an NQO1-dependent necrosis. Free Radic. Biol. Med. 166, 104–115. 10.1016/j.freeradbiomed.2021.02.014 33600944

[B187] Zhou B. Hao Q. Liang Y. Kong E. (2022). Protein palmitoylation in cancer: molecular functions and therapeutic potential. Mol. Oncol. 17, 3–26. 10.1002/1878-0261.13308 36018061 PMC9812842

[B188] Zmuda F. Chamberlain L. H. (2020). Regulatory effects of post-translational modifications on zDHHC S-acyltransferases. J. Biol. Chem. 295, 14640–14652. 10.1074/jbc.REV120.014717 32817054 PMC7586229

[B189] Zou C. Ellis B. M. Smith R. M. Chen B. B. Zhao Y. Mallampalli R. K. (2011). Acyl-CoA:Lysophosphatidylcholine acyltransferase I (Lpcat1) catalyzes histone protein O-palmitoylation to regulate mRNA synthesis. J. Biol. Chem. 286, 28019–28025. 10.1074/jbc.M111.253385 21685381 PMC3151047

